# Development of a Robust Method for Isolation of Shiga Toxin-Positive *Escherichia coli* (STEC) from Fecal, Plant, Soil and Water Samples from a Leafy Greens Production Region in California

**DOI:** 10.1371/journal.pone.0065716

**Published:** 2013-06-06

**Authors:** Michael B. Cooley, Michele Jay-Russell, Edward R. Atwill, Diana Carychao, Kimberly Nguyen, Beatriz Quiñones, Ronak Patel, Samarpita Walker, Michelle Swimley, Edith Pierre-Jerome, Andrew G. Gordus, Robert E. Mandrell

**Affiliations:** 1 Produce Safety and Microbiology Research Unit, United States Department of Agriculture-Agricultural Research Service, Albany, California, United States of America; 2 School of Veterinary Medicine and Western Institute for Food Safety and Security, University of California Davis, Davis, California, United States of America; 3 National Clonal Germplasm Repository for Citrus & Dates, United States Department of Agriculture-Agricultural Research Service, Riverside, California, United States of America; 4 Department of Biology, University of Washington, Seattle, Washington, United States of America; 5 California Department of Fish and Game, Fresno, California, United States of America; U. S. Salinity Lab, United States of America

## Abstract

During a 2.5-year survey of 33 farms and ranches in a major leafy greens production region in California, 13,650 produce, soil, livestock, wildlife, and water samples were tested for Shiga toxin (*stx*)-producing *Escherichia coli* (STEC). Overall, 357 and 1,912 samples were positive for *E. coli* O157:H7 (2.6%) or non-O157 STEC (14.0%), respectively. Isolates differentiated by O-typing ELISA and multilocus variable number tandem repeat analysis (MLVA) resulted in 697 O157:H7 and 3,256 non-O157 STEC isolates saved for further analysis. Cattle (7.1%), feral swine (4.7%), sediment (4.4%), and water (3.3%) samples were positive for *E. coli* O157:H7; 7/32 birds, 2/145 coyotes, 3/88 samples from elk also were positive. Non-O157 STEC were at approximately 5-fold higher incidence compared to O157 STEC: cattle (37.9%), feral swine (21.4%), birds (2.4%), small mammals (3.5%), deer or elk (8.3%), water (14.0%), sediment (12.3%), produce (0.3%) and soil adjacent to produce (0.6%). *stx1*, *stx2* and *stx1/stx2* genes were detected in 63%, 74% and 35% of STEC isolates, respectively. Subtilase, intimin and hemolysin genes were present in 28%, 25% and 79% of non-O157 STEC, respectively; 23% were of the “Top 6″ O-types. The initial method was modified twice during the study revealing evidence of culture bias based on differences in virulence and O-antigen profiles. MLVA typing revealed a diverse collection of O157 and non-O157 STEC strains isolated from multiple locations and sources and O157 STEC strains matching outbreak strains. These results emphasize the importance of multiple approaches for isolation of non-O157 STEC, that livestock and wildlife are common sources of potentially virulent STEC, and evidence of STEC persistence and movement in a leafy greens production environment.

## Introduction

Between 1982 and 2002, 350 outbreaks of *Escherichia coli* O157:H7 were reported in the United States; 52% and 9% were caused by foodborne or waterborne sources, respectively [1]. In addition to *E. coli* O157:H7, non-O157 Shiga toxin-producing *E. coli* (STEC) result in an average of 4,000 illnesses per year in the United States. However, even though non-O157 STEC disease has been reportable since 2000, the actual incidence of illness due to non-O157 STEC is unclear. Non-O157 STEC cases account for a substantial portion of all STEC infections [2], [3], [4], [5] with O-antigen types O26, O45, O103, O111, O145, and O121 responsible for most clinical cases of non-O157 STEC [6], [7]. However, many laboratories do not routinely isolate non-O157 STEC, primarily because of the lack of media and or immunochemical reagents for selecting, concentrating and identifying the non-O157 STEC O-types considered the most relevant clinically. Unlike most *E. coli* O157:H7 strains, non-O157 STEC contain glucuronidase and ferment sorbitol, factors exploited for detection of O157:H7 on indicator agar media. Since non-O157 STEC cannot be distinguished easily from other *E. coli* on these media, detection depends primarily on the ability to detect production of Shiga toxin (Stx) [8] or *stx* genes [9], [10].

Fresh leafy greens have been associated with multiple STEC infections [11]. Consumption of fresh fruits and vegetables is growing in the U.S. and this trend appears to correlate with an increase in produce-associated outbreaks. Contamination of produce could occur pre-harvest by application of raw (or poorly composted) manure, contaminated water (irrigation or flooding) [12], [13], [14], [15], or deposition of feces by livestock or wild animals. The presence of STEC anywhere in, or even near, a raw produce production environment must be considered a potential risk factor for human illness, both sporadic- and outbreak-related. Indeed, recent outbreaks involving O145 and O104:H4 STEC resulted in more than 50 (romaine lettuce, April – May 2010) and >4000 (Fenugreek sprouts, May – June 2011) reported illnesses, respectively, in addition to a significant incidence of hemolytic uremic syndrome (HUS) [16], [17]. The outcomes of the *E. coli* O104:H4 outbreak have raised questions about the emergence of new types of non-O157 STEC (enterohemorrhagic and enteroaggregative hybrid) with equal or greater virulence compared to O157:H7 strains, unusual clinical findings in some patients (e.g. neurological effects), and the mechanisms of pathogenesis in the absence of some of the recognized virulence factors (e.g. intimin, Tir, enterohemolysin) [17], [18].

An important objective of our multi-year survey of a major produce production region was to identify possible sources of STEC, especially O157, with the intent of tracking transport relevant to produce contamination. Therefore, we developed an isolation method designed to recover as many different STEC as possible from a variety of types of agricultural samples. Multiple suspect STEC colonies were recovered from each sample based on either colony color or morphology. Since some STEC strains will not grow, or not well, on selective chromogenic media, modified sheeps blood agar (mSBA) was incorporated at the latter period of our survey for isolating these STEC. Thus, our method for robust isolation of O157 and non-O157 STEC evolved during a large survey of a leafy greens production environment to a final method involving non-selective enrichment, separation of both O157 and non-O157 STEC on anti-O157 immunomagnetic beads (IMS), plating beads on two types media made for O157 isolation and on a non-selective medium, and finally, direct culture of PCR *stx*-positive enrichment broths on a third medium made for O157 isolation. Characterization of multiple STEC colonies from each of the large number of samples confirmed the value of using multiple selective and non-selective approaches for isolation of STEC.

## Materials and Methods

### Ethics Statement

To recruit participants in the study, we requested voluntary permission from growers and livestock operations to allow us to confidentially collect samples (lettuce/spinach, soil, water, sediment, animal feces) for the duration of the study. Participants were enrolled from private produce farms and ranches in Monterey, San Benito, San Luis Obispo Counties in the central California coast. For wildlife sample collection on private land, permission was obtained from owners enrolled in the study for USDA Wildlife Services (WS) or California Department of Fish and Game (DFG) personnel to enter the property and hunt/trap permitted species. Field crew notified and confirmed sampling dates with owners at least 24 hours before visiting the farm. Biosecurity to prevent cross-contamination between properties was ensured by field crew standard operating protocols for disinfection of all vehicles, equipment, and clothing/boots between visits. Field crew blinded private property identifying data (e.g., owner name, farm name, address, etc.) from the laboratory by using an 8-digit alpha-numeric code assigned to each location.

Wildlife sampling at all locations was approved under a set of California Department of Fish and Game (CDFG) Scientific Collection Permits issued to USDA Wildlife Services and CDFG personnel contracted to collect the samples and ship to USDA in Albany, California. Additionally, a federal permit with the U.S. Fish and Wildlife Services was obtained for sampling of geese, crows, and blackbirds. State and federal permits allowed hunting or trapping followed by humane lethal (sodium pentobarbital, shooting, and carbon dioxide) or non-lethal (capture-release) restraint methods appropriate for the targeted species. No endangered or listed species were included in the study. Because the wildlife sampling was conducted through a contract with state and federal wildlife agencies using their standard protocols, an Institutional Animal Care and Use Protocol was not submitted.

In addition to the enrolled farms and ranches, a subset of wildlife "convenience" samples were collected by USDA Wildlife Services personnel under state cooperator permits. These samples were collected during routine depredation work on private land in Monterey and San Benito Counties.

### Collection of Samples

As part of a survey of a major leafy greens production region in California, 13,668 samples of soil, livestock and wildlife feces, produce (predominantly leafy greens), water and watershed sediment were collected from 4/28/2008 until 10/26/2010. Samples were obtained using either clean latex exam gloves or single-use sterile spatulas. Soil samples were collected down to 5 cm from the soil surface. Plant samples were collected either as a single head of mature lettuce, or as a composite sample of multiple heads of younger plants. Heads were broken off at the stem, avoiding soil and discarding decayed leaves. Water samples were mostly collected from watersheds at public locations, but also from holding ponds, watering troughs, irrigation sources. An approximate 250 mL sample was collected in a sterile bottle using a telescoping pole at watershed sites to access, when possible, flowing water. However, many of the watershed sites with public access were sampled using Moore swabs anchored in flowing water for usually three days, as described previously [19]. The majority of the cow fecal samples were obtained by collecting the top portion of a fresh fecal pat, taking care to avoid pasture dirt underneath the feces. Additional cow fecal samples were collected by rectal grab. Approximately 30–35 fecal samples were obtained per sampling date on cattle ranches. Feces, plus part of the colon, were collected routinely from large wild mammals (feral pigs, coyotes, rabbits, opossum, raccoon) or crows by necropsy after hunting or trapping the animals in the field. Live small birds and mammals were trapped and sampled by obtaining a swab of the cloacal/anal area and then released. Swabs were stored in Cary Blair transport media (Fisher Scientific, Waltham, MA) and shipped for overnight delivery. Wildlife feces were collected from the ground, occasionally, when it was the only available source. All samples were stored and transported on ice in marked Whirl-Pak bags (Nasco, Modesto, CA). Samples were processed routinely within 24–48 hours after collection. A small number of fecal samples had a processing delay of 3–4 days after collection.

**Figure 1:**
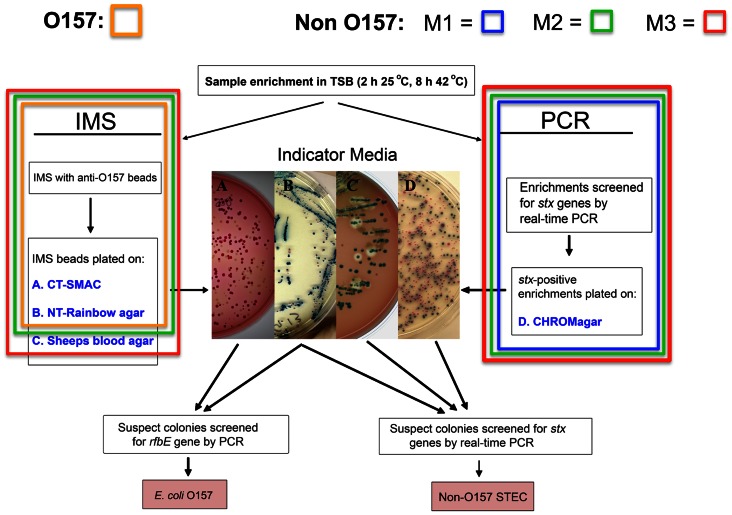
Flow chart for STEC isolation (O157 and non-O157) and examples of typical colony morphologies. The final isolation protocol (M3) incorporates the M1 and M2 methods and starts with enrichment in TSB and plating anti-O157 magnetic beads on three different media (“IMS”; media A, B, C) and direct plating of *stx*-positive enrichment broths on C-O157 (“PCR”; medium D). O157 suspect colonies appear as pale and steel blue colonies on SMAC and NT-RA, respectively. Suspect STEC colonies from any media are subcultured on LB and confirmed as either O157 or non-O157 STEC by PCR. Anti-O157 magnetic beads bind other bacteria present in enrichment broths of environmental samples, but, fortuitously, also many non-O157 STEC. Typical non-O157 STEC colonies are shown from enrichments growing on C-O157 (Indicator Media, panel D, blue colonies), NT-RA agar (panel B, pink colonies). Non-O157 STEC colonies expressing beta-galactosidase and hemolysin are indicated by blue colonies with a clearing zone of hemolysis on mSBA (panel C). The parts of the final method for isolating O157 and non-O157 STEC are shown by an orange box (O157), blue box (M1), green box (M2) and red box (M3).

All samples in this study were processed for generic *E. coli* and for *E. coli* O157 by the same methods (described below) throughout the 2.5 year survey. However, to increase the robustness of our method for isolating non-O157 STEC, we modified our initial method twice during the study. This resulted in three sets of samples, collected over three sequential periods, cultured separately by three different methods (Figure 1). Thus, our initial method for isolating non-O157 STEC (designated M1,4/28/08 to 10/15/08) involved PCR of the TSB enrichment broth to detect *stx* genes, plating samples on Chromagar O157 that reached a threshold Ct value, and isolating suspect STEC colonies. The second isolation method (M2, 7/19/08 to 1/13/10) continued theM1method and added the step of selecting suspect STEC colonies from the same Rainbow Agar plate where suspect O157 STEC were isolated. Finally, the third isolation method (M3, 1/8/10 to 10/26/10) continued the M2 method and added the step of selecting colonies from a modified sheep’s blood agar medium (mSBA).

### Measurement of Generic *E. coli* in Feces and Soil

The methods for quantifying generic *E. coli* were the same throughout the study and are separate from M1, M2 and M3 for STEC described in detail below. Ten g of feces, or one anal/cloacal swab, were added to 90 mL or 50 mL of Tryptic Soy Broth (TSB; Becton Dickinson, Sparks, MD), respectively, and agitated in a Pulsifier (Microgen Bioproducts, Surrey, UK) at maximum speed for 15 sec. Ten g of a soil sample were added to 90 mL of TSB and vigorously agitated by hand. One mL was removed from each sample for generic *E. coli* enumeration and the remainder used for sample enrichment as described below. One mL of 10-fold serial dilutions were plated on *E. coli*/Coliform Petrifilm (3 M Corp., St. Paul, MN) and incubated at 37°C for 24 hours. Generic *E. coli* colonies were recorded as the number of blue colonies with and without gas bubbles.

### Measurement of Generic *E. coli* in Water Samples and Moore swab and Plant Rinsates

Moore swabs from water were agitated vigorously in 350 mL of sterile water. Plant samples were tested initially by addition of 100 mL of sterile water to a 25 g plant sample. Subsequently, we changed the method by adding 350 mL sterile water to a 250 g sample. The samples were agitated vigorously, and the rinsate was transferred into a clean WhirlPak bag. One hundred mL of Moore swab or plant rinsates, or 100 mL of a batch water sample, were sealed in a QuantiTray 2000 tray containing Colilert reagent (IDEXX Laboratories, Westbrook, Maine) and trays were incubated at 37°C for 24 hr. The remainder of the rinsates was enriched in culture medium, as described below. The most probable number (MPN) of *E. coli* per 100 mL, per g of plant sample, or per swab were determined by counting the number of fluorescing wells and calculating according to manufacturer instructions.

### Sample Enrichment by Culture in Non-selective TSB

A schematic of the final isolation method developed during the study is shown in Figure 1. Soil, water sediment, feces or colon tissue samples were removed by hand using clean exam gloves or with a sterile spatula, and 10 g were added into a WhirlPak bag on a scale containing 90 mL TSB. A small percentage of the samples was enriched also in Rapid Check broth (Strategic Diagnostics Inc., Newark, DE) and lactose broth (Difco, Fisher Scientific) for comparison against TSB (see Results). One hundred mL of batch water samples were combined with 11 mL of 10x TSB sterilized by passage through a 0.45 µm filter. Moore swabs were processed for generic *E. coli* as described above, then 25 mL of 10x TSB was added to the bag containing the Moore swab and remaining rinsate (approximately 250 mL). Plant samples were processed initially by adding 25 g of sample to 250 mL TSB and then subjected to an enrichment step. Subsequently, the plant sample method was changed by adding 350 mL sterile water to a 250 g plant sample in a one gallon bag, agitating vigorously and decanting the rinsate into a WhirlPak bag. A 100 mL sample of plant or Moore swab rinsate was processed for enumeration of generic *E. coli* (see above) and a 1/10 volume of 10X TSB was added to the remaining rinsate prior to incubation. The bags were incubated for 2 hrs at 25°C with shaking at 200 RPM (Infors Multitron shaker with temperature program control, Bottmingen, Switzerland). The incubator was programmed to increase temperature to 42°C for 8 hrs with shaking, then decrease to 4°C and hold without shaking until the next morning when culture, isolation and *stx*-PCR procedures were initiated. One mL of the sample enrichment was stored in 15% glycerol frozen at −80°C for future use and a portion of the remaining sample was processed as described below.

### Detection of *Stx* Genes by RT-PCR in Enrichment Broth Lysates

A one mL sample of cultured enrichment broth was centrifuged for 2 minutes at 10,000×G and the pellet was resuspended in 1 mL of sterile water. A 100 µL sample was transferred to PCR tubes and heated in a PCR cycler (BioRad, Hercules, CA) to 80°C for 5 min and 100°C for 20 min, and the tubes were centrifuged for 10 min at 4000 RPM to remove cell debris. The supernatant was saved for analysis by real-time (RT) PCR.

Sensitive detection of *stx* genes in TSB sample enrichment broths is an important step in our non-O157 STEC isolation methods. Examination of the published sequences of *stx*1 and *stx*2 variants in GenBank revealed conserved regions for designing RT-PCR primers and probes. The available sequences for *stx*1 included the alleles *stx*1a, *stx*1c and *stx*1d, which were 96% homologous. This facilitated design of single primer/probe for amplifying all three types (Table 1). In contrast, the greater diversity of *stx*2 types compared to *stx*1 required additional primer/probe sets, including a specific set for *stx*2f. Two other primer/probes were designed from conserved regions to amplify either *stx*2 or *stx*2c (designated stx2abc) or the remaining *stx*2 types (designated stx2ex). Additionally, the four primer/probe sets (stx1, stx2abc, stx2ex, stx2f) were designed as a real-time stx quadraplex method to improve throughput.

**Table 1:** Primers and probes.

Name	Sequence^a^	Position^b^
Stx1 forward	CATCGCGAGTTGCCAGAAT	803
Stx1 reverse	TCCCACGGACTCTTCCATCT	874
Stx1 probe	Q670-ATCTGATGATTTCCTTCTATGTGTCCG-BHQ2	825
Stx2abc forward	GGACCACATCGGTGTCTGTTATT	167
Stx2abc reverse	CCCTCGTATATCCACAGCAAAAT	234
Stx2abc probe	CFO560-CCACACCCCACCGGCAGT-BHQ1	192
Stx2ex forward	GAAACTGCTCCTGTTTATACGATGAC	616
Stx2ex reverse	CCGGAAGCACATTGCTGAT	697
Stx2ex probe	FAM-CCCCCAGTTCAGAGTGAGGTCCACG-BHQ1	675
Stx2f forward	CGCTGTCTGAGGCATCTCC	608
Stx2f reverse	TCCTCTGTACTCTGGAAGAACATTAC	708
Stx2f probe	CFR610-TTATACAATGACGGCTCAGGATGTTGACCTTACC-BHQ2	630
ompA forward^c^	CAGTGATGCGAATCTATGCCA**AGATAACACCTGGTACACTGG**	72
ompA reverse^c^	TGCCAATCGCATGCTTTGCCTA**TGTGTGCGTCACCGATGTTG**	517
seq ompAf	CAGTGATGCGAATCTATGCCA	NA
seq ompAr	TGCCAATCGCATGCTTTGCCTA	NA

a Fluorescent probe labels are Q670, Quasar 670; CFO560, CAL Fluor Orange 560; FAM, Carboxyfluorescein and CFR610, CAL Fluor Red 610. Quenchers are BHQ, Black Hole Quencher 1 and 2 (Biosearch, Novato, CA).

b Position is relative to the coding region of *stx*1, *stx*2A or *omp*A from EDL933.

c *omp*A forward and reverse sequences in **BOLD** are homologous to *omp*A.

A 5 µL sample of the supernatant of the enrichment broth lysate was analyzed for the presence of *stx* by adding 0.3 µM of each primer, 0.2 µM of each probe (Table 1) and 10 µL Environmental Master Mix (EMM, Life Tech./Applied Biosciences, Foster City, CA), followed by incubation in a MX3000P RT-PCR machine (Stratagene/Agilent, Santa Clara, CA) at 95°C for 10 min, 40 cycles of 95°C for 20 sec, and 60°C for 45 sec. The Cycle threshold (Ct) value was determined for each primer/probe set.

### Quadraplex PCR for Confirming *stx* in Suspect STEC Colonies

Cells collected by sterile toothpick from individual suspect non-O157 STEC colonies from any of the four indicator plates were transferred on Luria-Bertani (LB) agar plates (Fisher Scientific) by making a small patch on the agar mapped by a grid template. Cells remaining on the same toothpick were transferred to a PCR mixture and tested by the *stx*-quadruplex PCR method described above, but with 2.5 units AmpliTAQ gold, 1x supplied PCR buffer, 3 mM MgCl_2_, 200 µM dNTP, 300 nM each primer, 200 nM each probe final concentration in a 20 µL reaction volume. Colony lysates resulting in Ct values <20 were considered positive for *stx* genes; *stx*-positive colonies were single-colony purified, then retested by the *stx*-quadruplex PCR method. Colonies remaining positive were grown aerobically in LB broth for 24 hr, and 700 µL of the culture were removed and frozen in glycerol until further use. Also, cells in 100 µL of the culture were pelleted by centrifugation at 2000×g for 5 min, pellets were resuspended in 100 µL of HyPure™ molecular biology-grade water (HyClone Laboratories, Inc., Logan, UT) and incubated at 95°C for 20 min. Lysed cell debris was removed by centrifugation at 2000×g for 5 min and the supernatants were collected and frozen until further use.

### Culture Media Used for Isolation Methods M1, M2, M3

Four media were used during this study and are included in the final and current method in our laboratory for isolation of STEC (M3): (Figure 1, medium A) Sorbitol MacConkey agar (Difco Labs; Detroit, MI) containing cefixime (0.05 µg/mL; Invitrogen/Dynal) and tellurite (2.5 µg/mL; Invitrogen/Dynal) (CT-SMAC); (Figure 1, medium B) Rainbow Agar O157 (Biolog, Hayward, CA) containing novobiocin (20 µg/mL; Sigma-Aldrich) and tellurite (0.8 µg/mL; Invitrogen/Dynal) (NT-RA); (Figure 1, medium C) mSBA; and (Figure 1, medium D) Chromagar O157 (C-O157) (DRG International, Mountainside, New Jersey). mSBA was prepared by adding 50 mL of washed, defibrinated sheep’s blood (BioMerieux, Durham, NC) to 1 L of sterilized BBL Blood Agar Base (Becton Dickinson, Sparks, MD) cooled to 45°C, and supplemented with 10 mM CaCl_2,_ 0.5 mg/L mitomycin C (Sigma-Aldrich, St. Louis, MO), and 50 mg/L X-Gal (Teknova, Hollister, CA), as described previously [20].

### Culture and Isolation of O157 STEC

**Figure 2:**
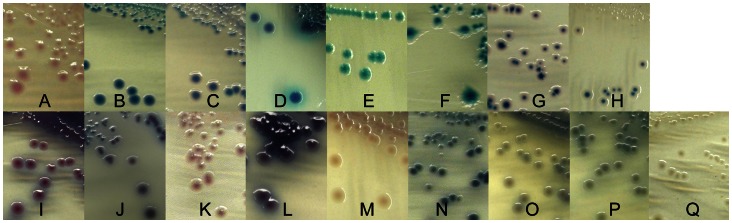
Colonies on C-O157 and NT-RA representative of colors and morphologies indicative of different STECs. Colony colors and morphologies of selected STEC are shown on C-O157 (A-H) and NT-RA (I-Q). Letter designations refer to Table 5. It was noted that colony color changed occasionally depending on the density of colonies (see C, H, and I).

*E. coli* O157:H7, and, fortuitously, non-O157 *E. coli* cells (see M2 and M3 details below), were captured immunochemically from 1 mL of each sample enrichment broth (Figure 1, “IMS”) by IMS with 20 µL of magnetic beads conjugated with anti-O157 antibody (Invitrogen/Dynal, Carlsbad, CA). For those enrichments where the amount of suspended material was high (for example, fecal samples), the sediment was removed by filtration (coffee filter) prior to IMS. The IMS was automated using the Dynal BeadRetriever (Invitrogen/Dynal, Carlsbad, CA) and the EPEC/VTEC protocol established by the manufacturer. IMS beads were resuspended and 50 µL were spread on two media: CT-SMAC and NT-RA. CT-SMAC and NT-RA plates with IMS beads were incubated at 37°C for 24 hours. Suspect O157:H7 colonies on CT-SMAC (colorless or light gray) and NT-RA (bluish gray) were selected by colony color and morphology (Figures 1 and 2). Suspect *E. coli* colonies were transferred by sterile toothpick into wells containing reagents for RT-PCR for the presence of the *rfb*E gene for O157 [21] or quadruplex RT-PCR for *stx* as described above.

### Methods for Culture and Isolation of Non-O157 STEC (M1, M2, M3)

We modified our initial method for isolation of non-O157 STEC (M1) twice during the study (M2, M3). However, it is important to note that each of the non-O157 methods included the O157 isolation method described above and run at the same time for every sample.

For M1 method, the enrichment samples were screened for *stx*1 and *stx*2 by RT-PCR (see above). Enrichment samples with Ct values below 27 for any of the 4 primer/probe sets were considered “positive,” and a 1–5 µl sample was streaked for single colony isolation on C-O157; the plates were incubated at 37°C for 18–24 hr. Suspect *E. coli* colonies (Figures 1 and 2) were transferred by sterile toothpick onto LB agar plates and into wells containing reagents for quadruplex RT-PCR for *stx*, as described above. *stx*-positive isolates were saved for additional characterization (see below).

The M2 method was performed as an addendum to M1, in that suspect non-O157 STEC colonies were selected based on color and morphology also from the same NT-RA plate used for isolating O157 (Figures 1 and 2), as suggested by the Biolog product insert. Suspect STEC colonies were transferred to LB agar and for RT-PCR for *stx,* as described above. *stx*-positive isolates were saved for additional characterization (see below).

The M3 method is the final and current method for isolation of O157 and non-O157 STEC and was performed as an addendum to the M2 method (Figure 1)**.** Briefly, 20 µL from the same anti-O157 magnetic beads sample used for the O157 isolation method were plated on mSBA. The plates were incubated at 37°C for 24 hr and putative STEC strains were identified by lysis surrounding the colony, indicative of one or more hemolysins.

### O-typing isolates by ELISA with Antisera Specific for 11 non-O157 STEC O-antigens

Rabbit antisera specific for O26, O45, O91, O103, O104, O111, O113, O118, O121, O145, and O146 were kindly provided by L. Cox, M. Erdman and B. Morningstar at USDA-APHIS National Veterinary Services Laboratory (Ames, IA); the anti-O104 antiserum was used only for analyzing a subset of isolates determined by PCR as O104-positive. An anti-O26 monoclonal antibody used for some assays was provided by J. Keen (U. Nebraska) [22]. For O-typing *stx-pcr positive* isolates, colonies were harvested from LB agar, suspended in 10 mM PBS, pH 7.4 to an OD_620_ of 0.2–0.3 (∼10^8^ cells/ml) and incubated at 55°C water bath for 30 min. 70 µl was dispensed in each of 12-wells in a single microtiter plate row (Maxisorp, Nalge Nunc Inc, Naperville, IL). The plates were dried overnight at 50°C. The wells were rinsed twice with distilled de-ionized (DD) water and 300 µl of blocking solution (1% BSA, 10 mM Tris-HCl, 150 mM NaCl, 5 mM MgCl_2_, 0.05% Tween 20, 30 mM sodium azide, pH 7.4) was added to each well and incubated for 1 hr, then rinsed with DD water. The antisera was diluted in 10 mM Tris-HCl, 150 mM NaCl, 1% BSA, 0.05% Tween 20, pH 7.4 (TBS-BSA) and 70 ul was added to each well in a single column on the plate, so that each isolate in the 12 well row was tested with all 12 antisera. The plate was incubated 1 hr (RT), and washed twice with Tris-buffered saline, 0.5% tween 20 followed by DD water. 70 uL of alkaline phosphatase-conjugated rabbit anti-mouse IgG (H+L) (Zymed, South San Francisco, CA) diluted 1∶1000 in TBS-BSA was added to each well and incubated 1 hr at RT. The wells were washed as described above and 70 µl of a 1 mg/ml p-nitrophenylphosphate substrate (Sigma, St. Louis, MO) diluted in 1 M diethanolamine, pH 8.0 was added to each well. The OD_405_ was measured after 30 min in an automated plate reader. An OD_405_ ranging from 1.5 to >3, depending upon the antisera, was designated as "putative positive" until confirmation by other methods. Selection from the same field sample was based on ELISA O-type pattern, *stx* type *(stx1-* and *stx2*-PCR), colony color on chromogenic media (Figure 2), and hemolysis on mSBA. These criteria minimized selecting identical strains from the same sample.

### Detection of O-antigen Type and Virulence Genes by PCR

A more complete characterization of thousands of STEC isolates identified during the study is ongoing. However, a subset of the strains (>250) was tested for O-type and virulence factors by PCR. *wzx* and *wzy* genes in the O-antigen gene cluster of 9 serogroups (O26, O45, O91, O103, O111, O113, O121, O145, and O157) were detected using PCR primers published previously [23]. All isolates were also screened by PCR to identify virulence genes encoding intimin (*eae*), subtilase (*subA*), enterohemolysin (*ehxA*), STEC autoagglutinating adhesin (*saa*), extracellular serine protease (*espP*), catalase peroxidase (*katP*), *stx1* (detecting all *stx1* variants), *stx2* (detecting all *stx2* variants), and the non-locus enterocyte effacement effectors (*ent/espL2* and *nleA*), as described in a previous report [23].

### MLVA for O157 and Non-O157 STEC

O157 STEC strains were analyzed by an 11-loci method described previously [21], [24]. Non-O157 STEC strains were analyzed by the 7-loci method described by Lindstedt et. al. for *E. coli* [25]. Briefly, three multiplex reactions with fluorescently labeled primers were used with boiled preps from overnight cultures to amplify seven fragments containing tandem repeats. Amplified fragments were size-fractionated by an ABI 3130 sequencer (Applied Biosystems) and allele numbers were assigned according to published methods and with MLVA algorithms in Bionumerics software (Applied Maths).

### ompA Sequencing

For *omp*A sequencing, primers were designed to conserved regions of the *omp*A gene, yielding approximately 501 bp, including 21 and 22 bp of additional sequence at the 5′ and 3′ ends, respectively, of both forward and reverse primers (Table 1). These additional sequences act as annealing sites for separate sequencing primers. The amplified fragment includes three of the first four transmembrane domains [26]. The *omp*A fragment was amplified from 1 µL of the same MLVA boiled preps and 2.5 unit AmpliTAQ gold, 1X PCR buffer, 1.5 mM MgCl_2_, 200 µM dNTP and 200 nM each primer in a 20 µL reaction. The reaction was run at 94° for 10 min, and 30 cycles of 94°C for 20 sec, 50°C for 30 sec and 72°C for 40 sec and a final extension reaction of 5 min at 72°C. Five µL of the above reaction was digested with 2 µL of ExoSAPIT (USB Corporation) at 37°C for 15 min and 80°C for 15 min. Big Dye Terminator reactions were run according to manufacturers protocol (ABI) using both forward or reverse primers. Unincorporated dye was removed by X-terminator (ABI) and the DNA fragments were resolved on an ABI 3730 sequencer. Sequences were aligned and trimmed using DNA Baser II software (Heracle Software).

### Phylogenetic and Statistical Analysis

Minimal spanning trees were constructed from 11-loci MLVA data from O157 STEC strains or from 7-loci MLVA data and *omp*A sequence data using BioNumerics Version 6.0. The MLVA similarity matrix was constructed using the Manhattan Distance algorithm and the UPGMA method. An *omp*A similarity matrix was prepared from the ompA sequence data using the Standard Algorithm and the default cost table. The similarity matrix from the non-O157 MLVA data was adjusted with a Distance Factor equal to 2.98. The Distance Factor was selected by comparison of the ranges of similarity values in the MLVA and ompA matrices to balance the contribution from each data set in the subsequent composite matrix. A composite similarity matrix was made by averaging the MLVA and ompA matices and by weighting the MLVA data over the sequence data 7∶1. This weight adjusted for the fact that MLVA data is 7 loci compared to 1 OmpA locus. Minimal spanning trees were constructed from the composite similarity matrix with N-locus variants weighted at 10,000 and 10 for N = 1 and N = 2, respectively. The highest scoring trees were selected after permutation resampling 1000 times.

Comparisons between sample treatments or correlations of incidence values were analyzed by t test or Pearson Correlation using Sigma Stat version 3.0 (SPSS, Chicago, IL). Most Probable Number (MPN) determination was used to predict the number of stressed cells in soil that had been contaminated with *E. coli* O157:H7 strain RM1484. The contamination level (as MPN) was determined by diluting the sample serially for five 2- fold dilutions (2.3 g to 92 mg), enriching 6 aliquots of each soil sample in TSB and processing them by O157-IMS as described in Methods. MPN was determined using the FDA/AOAC/BAM MPN Solver, Version 2002 [27]. Daily precipitation averages were computed from four weather sites in Monterey County from the California Weather Database (http://www.ipm.ucdavis.edu/) and designated as SALINAS, NSALINAS, GONZALAS, CASTROVL.

## Results

### Recovery of *E. coli* O157:H7 from NT-RA and CT-SMAC Media

Our priority goal at the initiation of the survey was to enhance efficiency of recovery of both *E. coli* O157:H7 and non-O157 STEC. An important aspect of our approach was to use an enrichment medium lacking antibiotics to enhance growth of sensitive STEC. We reasoned that harsh sample environments (surface water, feces from multiple animals with different microbiota, soil, plants) warranted a mild enrichment step to initiate resuscitation and growth. TSB enrichments of bird and cattle feces, water, soil and plant samples, that were determined to be STEC-negative by PCR, were inoculated with different concentrations of *E. coli* O157:H7 strain RM1484 (apple juice outbreak strain) and analyzed for sensitivity of recovery by IMS and plating on NT-RA and CT-SMAC. Additionally, soil samples inoculated with strain RM1484 cells and stored at room temperature for 1 month were also analyzed for efficiency of recovery of stressed cells. Recovery of strain RM1484 was verified by *rfb*E PCR of multiple colonies of the appropriate phenotype selected from the indicator media.

O157 STEC strain RM1484 inoculated at 56–78 CFU in *stx*-negative environmental sample TSB enrichments was detectable in all samples, including strain RM1484 incubated in soil one month (Table 2). However, the plant, water, soil and fecal swab samples were always positive at lower inoculum levels of 5–10 CFU per enrichment, whereas only 60–66% of the samples of feces were positive. We also tested the soil samples with strain RM1484 stored for 1 month in Rapid Check and Lactose enrichment broths for comparison to TSB. TSB and Rapid Check broth were similar at supporting growth of *E. coli* O157:H7 strain RM1484, but more effective than Lactose Broth, especially at low inoculum levels. Also, samples enriched for 20 hr in TSB and Rapid Check broth resulted in no significant improvement in recovery compared to the 8 hr enrichment (Table 2). Because TSB provided effective enrichment and was of reasonable cost, it was used in all subsequent experiments and survey sample testing.

**Table 2:** Sensitivity of three enrichment methods for recovery of *E. coli* O157 from environmental samples.

		Fraction of positive samples	
Sample type	Incubation conditions	5–10 CFU per enrichment^b^	56–78 CFU per enrichment^b^
Bird (mallard), cloacal swab	2+8 hr^a^ in TSB	6/6	6/6
Bird (mallard), feces	2+8 hr in TSB	**4/6**	6/6
Cattle feces	2+8 hr in TSB	**12/20**	20/20
Water	2+8 hr in TSB	20/20	20/20
Soil	2+8 hr in TSB	16/16	16/16
Plants (Romaine lettuce), 25 g	2+8 hr in TSB	22/22	22/22
Plants (Romaine lettuce), 250 g	2+8 hr in TSB	6/6	6/6
Soil, stressed inoculum	2+8 hr in TSB	14/14	14/14
Soil, stressed inoculum	2+20 hr^c^ in TSB	14/14	14/14
Soil, stressed inoculum	2+8 hr in lactose broth	6/6	6/6
Soil, stressed inoculum	2+20 hr^c^ in lactose broth	**5/6**	6/6
Soil, stressed inoculum	2+20 hr^c^ in Rapid Check broth	**5/6**	6/6

a 2 hr incubation at 25**°**C and 8 hr at 42**°**C with shaking.

b Inoculum was *E. coli* O157 RM1484. Inoculum level was determined by serial plating on LB. <100% of samples positive are shown in **boldface** type.

c 2 hr incubation at 25**°**C and 20 hr at 42**°**C with shaking.

### Development of Quadraplex RT-PCR for *stx1* and *stx2* Subtype Genes

A preliminary test of the effectiveness of the *stx* multiplex PCR primer set (Table 1 and Methods) was by amplification of *stx1, stx1c, stx2, stx2b, stx2c, stx2d, stx2d, stx2e* and *stx2f* present in one or more of a set of 48 STEC isolates representing 14 different O-types and obtained from the *E. coli* Reference Center at Penn State University (Table S1). The multiplex PCR primer sets amplified all strains containing *stx1* or *stx2* correctly. Additionally, the multiplex reaction amplified *stx1* fragments from two isolates (O76 STEC strain RM6932 and O128 STEC RM6960) and *stx2* fragments from one strain (O147 STEC RM6970) lacking *stx* variant details (Table S1). Similarly, the multiplex reaction amplified *stx*1 and *stx*2 from strains that were shown by Vero cell assay to produce Stx (O118 strain RM6954, O121 strain RM6955, O147 strain RM6971 and O147 strain RM6972), but lacked detection of a known Stx variant. These results indicated also that the stx2abc and stx2ex primer/probe sets measured specificities that overlapped significantly. Nearly all isolates of different *stx*2 types yielded low Ct values (i.e. high sensitivity) with both primer/probe sets. The two exceptions were isolates positive for *st*x2e and *stx2f*, which are more divergent than the other *stx*2 types. These isolates were amplified only with the stx2ex and stx2f primer/probe sets, respectively. The four primer/probe sets were used to analyze all sample enrichments processed in this study.

### Recovery of STEC by Non-selective Isolation on C-O157 Media

**Figure 3:**
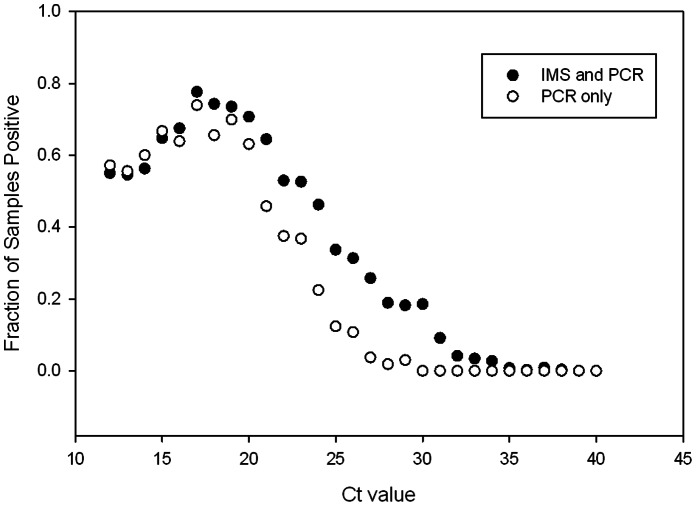
Recovery of non-O157 STEC strains from enrichment samples with different RT-PCR Ct values for *stx*. The fraction of samples positive for isolation of at least one non-O157 STEC strain based on threshold PCR Ct value (M1) is compared to the fraction positive with both the PCR and IMS methods (M2). A Ct <27 was selected as the value required for routine plating of an enrichment broth on C-O157. However, all enrichment broths, regardless of Ct value for *stx*, were treated with O157-IMS and beads were plated on NT-RA(IMS method) for attempted recovery of non-O157 STEC strains (M2).

Enrichments were screened for *stx* genes by multiplex RT-PCR and positive enrichments were plated on C-O157 (Figure 1, Panel D and Figure 2). As expected, the rates of recovery of non-O157 STEC from C-O157 were inversely proportional to the Ct values of the amplifications for *stx*. At least one strain of non-O157 STEC was recovered from 34% of the sample enrichment broths with Ct values, for any of the four primer sets, <27 and plated on C-O157; they were recovered from only 0.13% of the enrichments with Ct >27 for all four primer sets and 0.02% of enrichments with No Ct values (Figure 3). For a few samples, we attempted to isolate STEC by screening >100 suspect colonies from C-O157 plated with PCR Ct >27 enrichment broths, but only identified from 0 to 2 STEC isolates (data not shown). These results supported using a threshold value for plating on C-O157 and the addition of other methods not dependent on PCR [i.e. screening NT-RA (M2) and IMS with mSBA (M3)].

To evaluate the sensitivity of the multiplex RT-PCR for detecting *stx* in complex enrichments, microbiota in fecal, soil, plant or water samples were enriched, then inoculated with *E. coli* O157 strain RM1484 and analyzed by RT-PCR. The results indicated assay sensitivity of approximately 10^4^ CFU per reaction. Using a Ct value of 27 as our threshold for “positive” (i.e. Ct ≤27), the sensitivity of detection was approximately 3.5×10^6^ CFU of STEC per mL of enrichment (Table 3). To improve the sensitivity of our RT-PCR for measuring *stx* in complex samples, we compared detection of *stx* in enrichments of soil, water, lettuce and fecal samples inoculated with strain RM1484 with and without TaqMan Environmental Master Mix (EMM; Life Tech./ABI), a mix of reagents enhancing “pathogen detection in the presence of high levels of inhibitors.” EMM resulted in an improvement of sensitivity of at least one Ct unit (about 3 fold), with the most significant improvements with the most inhibitory samples of PCR, soil and fecal samples (Table 3).

**Table 3:** RT-PCR with and without Environmental Master Mix (EMM) to detect *stx* genes in environmental sample enrichments spiked with STEC.

Sample Type^a^	Treatment	Samples (N)	Average CFU/mL (X10^6^) at Ct 27 (SD)^b^
Soil	None	5	>10.7 (6.7)^B^
Soil	EMM	5	3.1 (3.0)^A^
Water	None	7	3.8 (3.2)^A^
Water	EMM	7	3.4 (3.2)^A^
Lettuce	None	3	3.1 (3.7)^A^
Lettuce	EMM	3	3.4 (4.0)^A^
Feces	None	6	>12.7 (7.6)^B^
Feces	EMM	6	3.5 (3.5)^A^

a Lettuce = Iceberg; Water = surface water; Feces = cattle feces. Plants and soil were collected from coded farm location F; Cattle feces were collected from coded ranch location Q. Surface water was collected locally (37.882855 N, 122.300007 W). All samples tested as STEC-negative prior to spiking.

b Enrichments were spiked with 10 fold dilutions of *E. coli* O157 strain RM1484 (0–20×10^6^ CFU/mL) and subjected to RT-PCR using AmpliTaq Gold or EMM (see Methods). Each of the samples produced a Ct, except for several soil and feces samples lacking a Ct even at the highest inoculum. A Ct of 27 corresponds to the upper limit we have set for culturing enrichment samples for isolation of STEC. SD, standard deviation. Different letter superscripts designate samples with a significant difference in treatment results (P<0.05).

### Recovery of Non-O157 STEC from NT-RA and mSBA Media

O157 STEC isolates were selected from IMS beads plated on NT-RA and CT-SMAC plates. However, we noticed that numerous suspect *E. coli* colonies of colors and morphologies (as described in RA product literature; Biolog, Hayward, CA) were present often on the same NT-RA plates and, especially, those from fecal samples (Figure 1, panel B and Figure 2). However, our goal to isolate any STEC, regardless of O-type or virulence type, stimulated us to modify the initial method for non-O157 STEC (“PCR Method” only; part of M1), by adding selection of suspect colored colonies observed on NT-RA (Figure 2) plated with O157-IMS beads (M1 plus this addition = M2). The benefit of including the “IMS-NT-RA” strategy is evident in Figure 3 at enrichments with Ct values between 21 and 31 corresponding to a higher fraction of samples tested by M2 as positive with IMS-NT-RA, and enrichments with Ct values between 30 to 34, corresponding to samples positive only with IMS-NT-RA. A method supporting this approach has been published recently [28]. Our final addition to our STEC method was plating the O157-IMS beads also on mSBA and selecting colonies with a zone of lysis (Figure 1, Panel C) according to methods published previously [20], [29]; M2 plus this addition designates M3.

### Recovery of O157 STEC by All Isolation Methods

**Figure 4:**
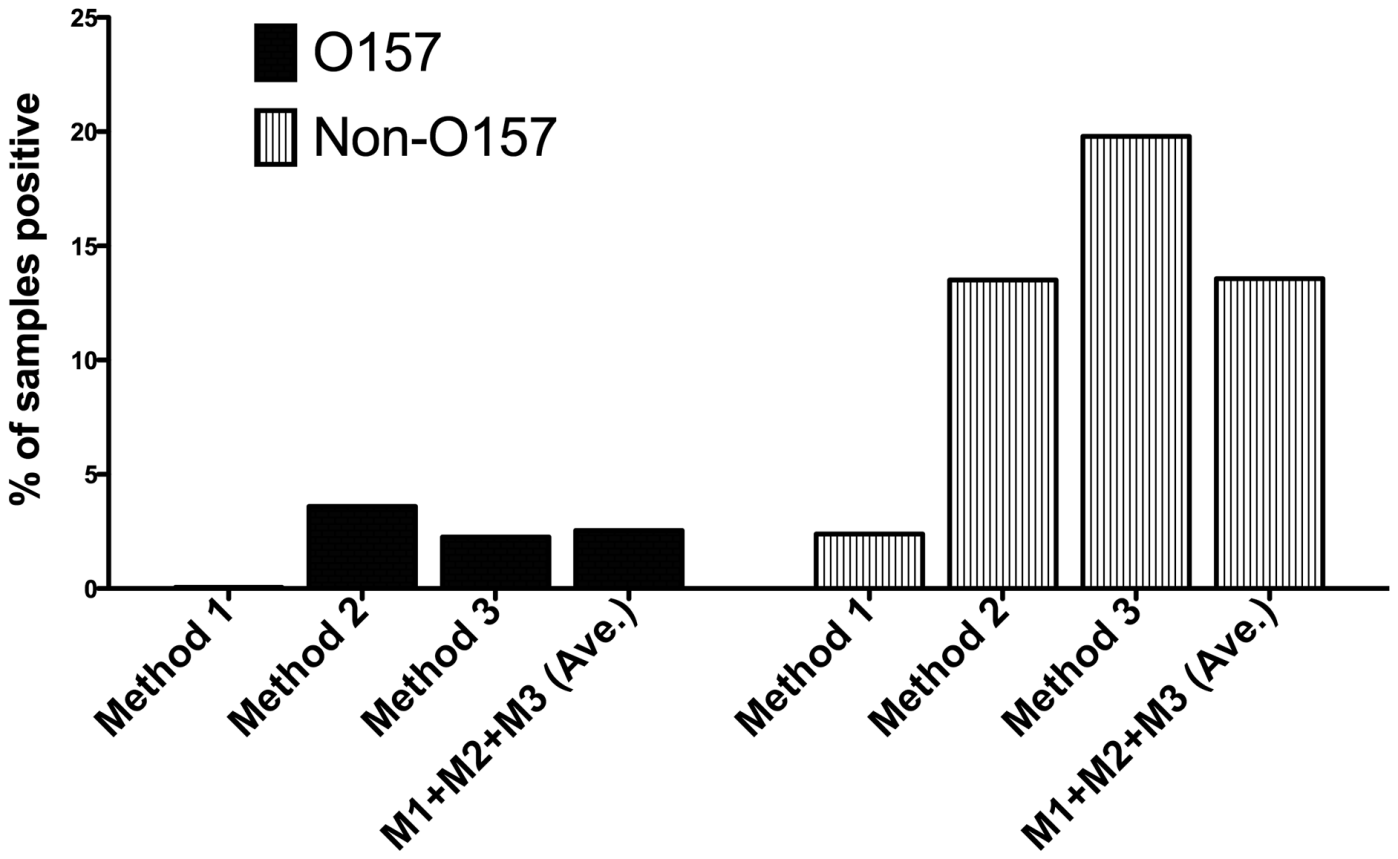
Incidence of O157 and non-O157 STEC in samples processed by M1, M2 and M3. Samples were processed over a 2.5 year period by M1 (IMS and NT-RA for O157 only+plating enrichment with Ct <26 for stx on C-O157), M2 (M1+ picking suspect STEC from NT-RA), M3 (M2+ mSBA).

During an approximately 2.5 year survey, 2133, 6977 and 4558 samples (total = 13,668) samples were processed by M1, M2 and M3, respectively. This resulted in 1 (0.05% of samples tested), 248 (3.6%) and 114 (2.5%) O157 STEC-positive samples (Figure 4). The O157 STEC results are comparable for all periods, because >99% were isolated by IMS-NT-RA, which was the same strategy for O157 in all 3 methods (occasionally, an O157 strain was isolated from C-O157 or SRBA, but in almost all cases, an O157 was isolated also from the same sample on NT-RA). Of the 363 O157 STEC-positive samples (2.7% of total samples), domestic ruminant samples were most often positive (6.6%), followed by sediment (4.0%), water from watersheds (4%) and ranches (3%), and wildlife (1.2%) (Table 4). Twenty-six of the 37 O157-positive wildlife samples were from feral swine (70.3%); 8 were from birds (2.1%), 2 from coyotes and 1 from tule elk. The 43 O157-positive water and water-sediment samples represented 23 from watersheds, 5 from ponds or irrigation ditches on a produce farm, and 12 from surface water or water trough well sources on a livestock ranch (Table 4). Only 1 soil sample from a produce farm was O157-positive. Nine additional O157-positive “soil” samples were from dry cattle ranch pasture soil.

**Table 4:** Comparison of samples positive for O157 STEC and non-O157 STEC by M1, M2 and M3 at corresponding sequential periods.

Sampling period(Method)	Source^a^	Samples (N)	O157 (N)	Non-O157 (N)	O157 and/or Non-O157 (N)	O157 (% )	Non-O157 (%)
**Apr–08 to Oct–08 (M1)**	Domestic ruminants	152	0	15	15	0.00	9.87
	Wildlife	410	1	28	29	0.24	6.83
	Soil	715	0	0	0	0.00	0.00
	Produce	712	0	1	1	0.00	0.14
	Water (ranch)	25	0	5	5	0.00	0.00
	Water (farm)^b^	57	0	0	0	0.00	0.00
	Water (watershed)	0	0	0	0	0.00	0.00
	Sediment	62	0	2	2	0.00	3.23
	All sources	2,133	1	51	52	0.05	2.39
**Jul–08 to Jan–10 (M2)**	Domestic ruminants	2,135	193	684	877	9.04	32.04
	Wildlife	1,593	23	115	138	1.44	7.22
	Soil	1,200	10^c^	11	21	0.83	0.92
	Produce	1,056	0	7	7	0.00	0.76
	Water (ranch)	220	7	36	43	3.18	16.36
	Water (farm)	158	0	13	13	0.00	8.23
	Water (watershed)	324	11	42	53	3.40	12.96
	Sediment	291	4	39	43	1.37	13.40
	All sources	6,977	248	948	1196	3.55	13.59
**Jan–10 to Nov–11 (M3)**	Domestic ruminants	1,677	70	730	800	4.17	43.53
	Wildlife	1,199	13	93	106	1.08	7.76
	Soil	535	0	0	0	0.00	0.00
	Produce	694	0	7^d^	7	0.00	1.01
	Water (ranch)	93	3	19	22	3.23	20.43
	Water (farm)^b^	29	0	7	7	0.00	24.14
	Water (watershed)	244	12	36	48	4.92	14.75
	Sediment	87	16	13	29	18.39	14.94
	All sources	4,558	114	905	1019	2.50	19.86
**Apr–08 to Nov–11 (M1+M2+M3)**	Domestic ruminants	3,964	263	1,429	1,692	6.63	36.05
	Wildlife	3,202	37	236	273	1.16	7.37
	Soil	2,450	10	11	21	0.41	0.45
	Produce	2,462	0	16^d^	16	0.00	0.65
	Water (ranch)	338	10	60	70	2.96	17.75
	Water (farm)^b^	244	0	20	20	0.00	8.20
	Water (watershed)	568	23	78	101	4.05	13.73
	Sediment	440	20	54	74	4.55	12.27
**Total**	All sources	13,668	363	1,904	2,267	2.66	13.93

a Domestic ruminant samples includes cattle, alpaca, goat. Soil, designates soil next to leafy green plants predominantly. Water (ranch), designates samples of standing water or trough water on a livestock ranch. Water (farm), designates irrigation, standing or creek/stream water on a leafy greens produce farm. Water (watershed), designates water from the Salinas River and tributaries and other watershed sites with public access.

b Water (farm), designates farms growing leafy greens.

c Nine of the 10 O157 positive samples were isolated from very dry soil collected in a cattle ranch pasture.

d Five of the non-O157 STEC-positive produce samples were identified by screening by M3 additional suspect colonies from a sample of the saved frozen TSB enrichment broths.

The average results presented in Figure 4 for comparison are by sampling period and method, but it should be noted that different numbers and types (e.g. sources, species, ranches/farms) of samples are represented in each period. For instance, the relatively poor O157 recovery by M1 may simply reflect the low number of cattle samples tested (n = 152) compared to M2 (n = 2000) and M3 (n = 1524) during this period.

### Recovery of Non-O157 STEC by All Isolation Methods at Corresponding Sampling Periods

Samples processed for non-O157 STEC occurred in three sequential periods corresponding to the addition of a second step (period 2/M2: isolation from O157 IMS NT-RA) and addition of mSBA (period 3/M3). Again, the modifications of methods were to increase the probability of isolating non-O157 STEC strains of any type. The results are presented by sampling period and method for comparison, but it is important to note that different numbers and types (e.g. sources, species, ranches/farms) of samples are represented in each period, precluding any strong statistically significant comparisons of the results between methods. Nevertheless, the incidence of non-O157 STEC increased with each modification to M1. M1, M2 and M3 resulted in 2.4%, 13.6% and 19.9% overall incidence of non-O157 STEC, respectively (average for all methods = 13.9%) (Table 4 and Figure 4). Domestic ruminants had the highest incidence of STEC increasing from 9.9% with M1 to 32.0% and 43.5% incidence with M2 and M3, respectively. Wildlife, water and sediment samples processed by M2 and M3 resulted in significant incidence of non-O157 STEC ranging from 7.2% to 24%.

We processed 4,507 samples by our final prototype method (M3) involving RT-PCR for *stx*, plating *stx*+ enrichments (Ct <27) on C-O157 and plating O157-IMS beads on both NT-RA and mSBA. Of the samples processed by M3, feces and cloacal swabs represented 63.6% (2867/4507) of the samples; approximately 58.5% of the fecal samples were from domestic ruminants and the remainder were from wildlife. The remaining samples were 694 leafy green produce (15.4%), 535 soil (11.9%), 315 water (7.0%) and 87 sediment (1.9%) samples. Overall incidences of non-O157 STEC by M2 and M3 were much higher than by M1 (13.6 and 19.9 versus 2.4, respectively), and incidence by M3 was higher for several sample types (domestic ruminants, water samples, sediment) compared to M1 and M2.

### Colony Color and Morphology on NT-RA and C-O157 are Indicators of Non-O157 STEC

Colonies of different colors and morphologies were observed on indicator media, as noted in manufacturer-provided product information. Multiple STEC strains of 8 O-types in our collection were monitored for colony color on C-O157 and NT-RA (Figure 2 and Table 5). All of the STEC O45 and O91 strains tested, and several O113 and O121 strains, failed to grow on NT-RA. The O-types assessed had characteristic colony color, including *E. coli* O157 strains. However, the predominant colony color for an O-type was not displayed uniformly on C-O157, often due to colony density on the plates. These changes involved different colors on the perimeter of the colony (O26, O45, O91, O103, O121), in colony centers (O111), and sometimes morphology differences (e.g. halos for O45) or swarming appearance (O113). In contrast, strain growth and colony morphology were more uniform on NT-RA. STEC O103 and O111 did not vary on NT-RA. Despite the predominant colony color for each O-type, a few strains of STEC O26, O113, O121, O145 and O157 displayed different colors on these media and were confirmed as specific O-types (Figure 2 and Table 5).

**Table 5:** Comparison of O-types and colony colors among STEC.

				Number of isolates by O-type^a^								
Indicator agar	Colony morphology		O26		O45	O91	O103	O111	O113	O121	O145	O157
C-O157	pink	A^b^	1		0	0	0	0	0	0	0	**37**
C-O157	blue w/white perimeter	B	**40** c		0	**5**	0	0	**18**	**5**	0	0
C-O157	blue w/pink perimeter	C	1		0	0	**17**	0	0	0	0	0
C-O157	blue, white perimeter, blue halo	D	0		**10**	0	0	0	0	0	0	0
C-O157	turquoise	E	0		0	0	0	0	1	1	4	0
C-O157	bluish turquoise, swarmy	F	0		0	0	0	0	6	0	0	0
C-O157	pink w/blue center	G	0		0	0	0	**12**	0	0	0	1
C-O157	white w/blue center	H	0		0	0	0	0	0	0	**7**	0
NT-RA	purple	I	**38**		0	0	0	0	0	0	4	0
NT-RA	bluish purple	J	0		0	0	0	0	0	0	0	1
NT-RA	pink	K	1		0	0	0	0	6	2	2	0
NT-RA	violet	L	1		0	0	0	0	0	0	0	0
NT-RA	grayish pink	M	0		0	0	**17**	0	0	0	0	0
NT-RA	steel gray	N	0		0	0	0	0	0	0	0	**35**
NT-RA	grayish purple	O	1		0	0	0	0	0	0	**7**	0
NT-RA	grayish blue	P	1		0	0	0	**12**	0	0	0	0
NT-RA	white w/gray center	Q	0		0	0	0	0	0	0	0	2
NT-RA	no growth		0		**10**	**5**	0	0	**19**	**4**	0	0
Total number			42		10	5	17	12	25	6	11	38

a Serotyped STEC isolates were streaked for single colonies on C-O157 or NT-RA and colony morphology of each were categorized into groups based on both color and colony shape.

b Letter designation refers to Figure 2.

c Boldface numbers indicate most common colony characteristics for each O-type on the indicator agar.

### Identification of Virulence Genes in STEC Isolates

All of the O157 and non-O157 STEC isolates were characterized by PCR for a set of genes correlated with virulence (Table 6). The *stx*2 gene was more frequent than *stx*1 in both O157 (97% to 40%) and non-O157 STEC (74% to 63%), but 37% and 35% of O157 and non-O157 STEC isolates contained both *stx*1 and *stx*2, respectively. Hemolysin genes (*hlyA* in non-O157 or *ehxA* in O157) were detected frequently in O157 (99%) and non-O157 (79%) isolates. Intimin (*eae*) was present in 100% of the O157 insolates, but in only 25% of the non-O157 isolates. Also, *eae* was present in only 8.2% of the STEC isolates from produce samples (all were non-O157) compared to ≥23% for all other sample types (>1.5 SD from the mean). Comparison of non-O157 isolates from other domestic ruminant and wildlife feces indicated no significant differences in incidence of these virulence genes.

**Table 6:** Incidence of virulence genes in all O157 and non-O157 STEC isolates^a^.

Number of O157 STEC isolates (% of total)								
Sample source	Total recovered	*stx*1	*stx*2	*stx*1/*stx*2^b^	*fliC*	*eae*	*hly*A	
Domestic ruminants^c^	541	200 (37)	522 (96)	181 (33)	541 (100)	54 (100)	541 (100)	
Wildlife (feces)	40	22 (55)	40 (100)	22 (55)	40 (100)	40 (100)	40 (100)	
Wildlife (swabs)	12	0 (0)	12 (100)	0 (0)	12 (100)	12 (100)	12 (100)	
Soil	5	5 (100)	5 (100)	5 (100)	5 (100)	5 (100)	2 (40)	
Produce	0	0 (0)	0 (0)	0 (0)	0 (0)	0 (0)	0 (0)	
Water^d^	60	35 (58)	60 (100)	35 (58)	60 (100)	60 (100)	59 (98)	
Sediment	12	8 (67)	12 (100)	8 (67)	12 (100)	12 (100)	12 (100)	
Total	670	270 (40)	651 (97)	251 (37)	670 (100)	670 (100)	666 (99)	
**Number of Non-O157 STEC isolates (% of total)**								
**Sample source**	**Total recovered**	***stx*** **1**	***stx*** **2**	***stx*** **1/** ***stx*** **2** a	***sub*** **A**	***eae***	***ehx*** **A**	
Domestic ruminants	2,590	1,605 (62)	1,944 (75)	962 (37)	754 (29)	577 (23)	2,103 (82)	
Wildlife (feces)	304	246 (81)	245 (80)	97 (32)	91 (30)	76 (25)	219 (72)	
Wildlife (swabs)	49	13 (26)	47 (96)	5 (10)	5 (10)	26 (53)	18 (37)	
Soil	20	5 (25)	19 (95)	4 (20)	3 (15)	5 (25)	8 (40)	
Produce	60	25 (41)	47 (77)	12 (20)	34 (56)	5 (8.2)	48 (79)	
Water	187	120 (64)	101 (54)	36 (19)	29 (16)	94 (50)	150 (80)	
Sediment	74	62 (84)	46 (62)	34 (46)	15 (20)	28 (38)	63 (85)	
Total	3,284	2076 (63)	2449 (74)	1150 (35)	931 (28)	811 (25)	2609 (79)	

a Represents isolates saved after screening a larger number of isolates by stx-PCR, O-typing ELISA, MLVA and ompA sequencing. Data from this characterization facilitated selection of isolates to represent those from an individual sample and minimize replication of strains from a sample. STECs isolated by all 3 methods are included.

b Both *stx*1 and *stx*2 present in the same strain.

c Cattle, alpaca, goats.

d Isolates from any water sample are included.

### Factors Affecting Recovery of STEC

The data obtained from processing thousands of samples during a 2.5 year period revealed a number of factors associated with the sensitivity of recovery of STECs. For example, overnight courier transport of samples from Salinas field sites to our lab (a distance of approximately 160 km) for sample processing occasionally took 1–3 days longer than the 1 day for the majority of samples. All soil, produce and water samples were processed one day after sample collection. In contrast, 484 (11%), 16 (0.4%) and 3 (0.07%) fecal samples were processed 2, 3 or 4 days after sampling, respectively. The 6% and 27% of the total samples positive for *E. coli* O157 or non-O157 STEC, processed one day after sampling, was significantly greater than 0.6% and 14% of samples delayed in processing and positive for *E. coli* O157 or non-O157 STEC, respectively, (P<0.001). We processed some samples after similar storage/shipping conditions in the laboratory by inoculating *E. coli* O157 at 4 CFU into 15 STEC-negative cattle fecal samples (negative for STEC by PCR and culture), holding samples on ice for 0, 1, 3 and 7 days and processing by the IMS isolation method. *E. coli* O157 was recovered from 86, 93, 60, and 66% of the samples, respectively, indicating an approximately 30% loss of viability and/or recovery with >1–2 days delay in sample processing.

### Culture Bias

**Figure 5:**
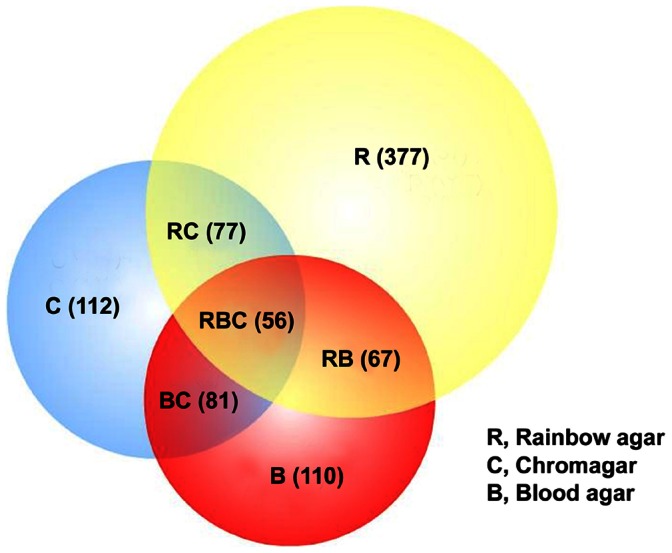
Venn diagram of samples positive for non-O157 STEC by NT-RA, C-O157 and mSBA. The enrichment broths for 4,160 samples (sampling period Jan – Oct 2010) were processed by M3 corresponding to plating on C-O157 (PCR method; “C”), NT-RA agar (IMS method; “R”) and mSBA (IMS method; “B”). The figure shows the number of samples that were positive for non-O157 STEC on only 1 of the 3 media (R, C, B) and any combination of the 3 media (BC, RB, RC, RBC).

Our method for isolating non-O157 STEC involved parallel procedures designated as “PCR method” (with C-O157) and “IMS method” (with both NT-RA and mSBA) (Figure 1). Although the three methods (M1, M2, M3) were not tested with the same samples obtained at the same time, samples processed by M3 provided comparison of the efficiency of isolation of non-O157 STEC from the same samples on the three media yielding STEC. The data summarized in Figure 5 illustrate that 377, 112 and 110 samples were positive only on NT-RA, C-O157 and mSBA, respectively. Only 56 samples were positive on all 3 media (Figure 5, “RBC”) and <90 of the 4160 samples tested on all 3 media were positive for STEC on any 2 media (Figure 5: “BC” = 81, “RB” = 67, “RC” = 77). The results indicating possible differences in the fitness of some strains on different media (“culture bias”).

### Virulence Genes

**Figure 6:**
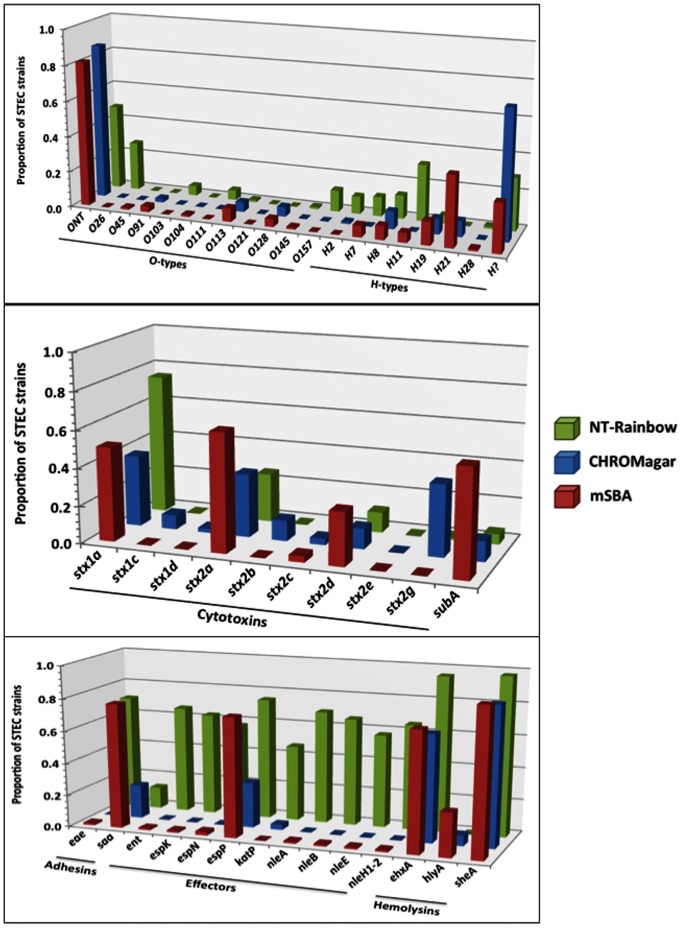
Virulence genes and O-antigen genes in a subset of STEC strains. A subset of strains isolated by the final prototype method (M3; all media) was analyzed by PCR as described previously (Quinones et al, 2012, Frontiers). This provided an opportunity to compare the types of strains isolated from samples exposed to all three media used for isolation of non-O157 STEC.

To determine the virulence profile of strains isolated from different media (Figure 5), O-types and virulence genes of STEC strains from each medium were assessed by PCR (Figure 6). O-types O26, O103, O111, O145 and O157 were isolated more frequently from IMS on NT-RA compared to the PCR method (no IMS, C-O157); conversely, O45, O91, O113 and O128 O-types were isolated more frequently from C-O157 and mSBA (P<0.001). Non-O157 STEC strains untypable by our typing assays (ELISA and PCR) were isolated at a similar proportion by the three methods. However, the most significant differences in strains from NT-RA, mSBA and C-O157 media were virulence gene incidence (Figure 6). Strains of the *stx* subtypes were detected at different proportions from RA, mSBA and C-O157 media (Figure 6). *stx1c*-, *stx2b*- and *stx2g*-positive strains were isolated only from C-O157, and *subA*-positive strains predominantly from mSBA. Strains positive for *eae* (“adhesion”), *ent*, *espK*, *espN*, *katP*, *nleA*, *nleB*, *nleE*, or *nleH1–2*, were isolated predominantly, or only, from NT-RA. The higher proportion of *hlyA*-positive strains from mSBA was consistent with blood hemolysis as a selection criterion. These results indicate a significant bias in the virulence profile of strains isolated from NT-RA, mSBA and C-O157 (Figures 5 and 6).

### Seasonal Incidence of O157 and Non-O157 STEC in Cattle, Feral Pigs and Water Samples

**Figure 7:**
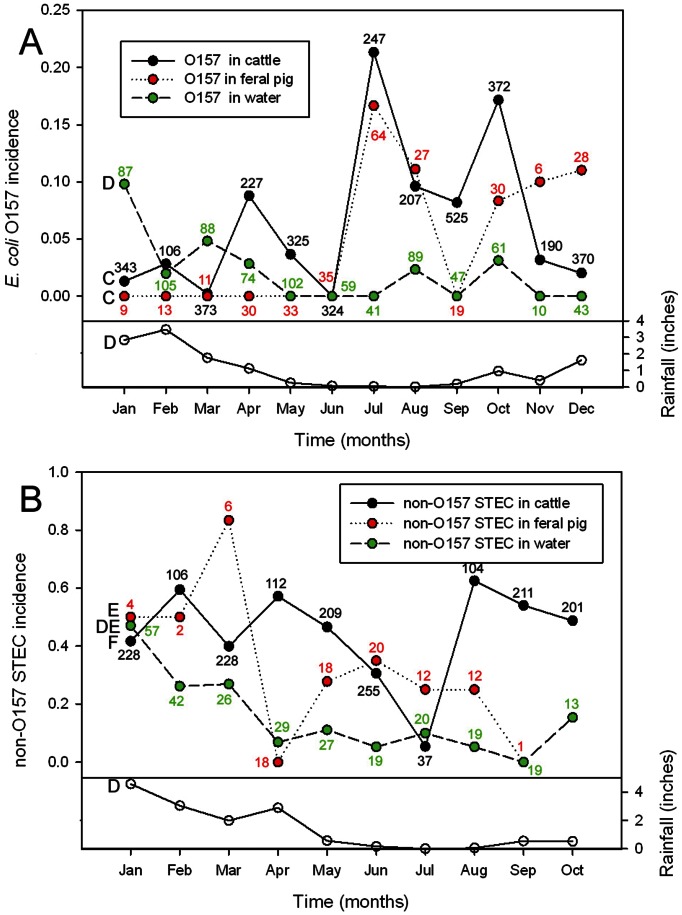
Seasonal isolation of O157 and non-O157 STEC from cattle, pig and water samples. The fractions of cattle, feral pig or water samples positive for at least one O157 (A) strain are shown for different months of the year. Each point represents the average fraction of positive enrichments, processed by the IMS method, for each month over a 30-month period from April 2008 until October 2010. Numbers adjacent to each point are the number of samples represented. Monthly rainfall averages are the average of 4 weather sites during the 30 month period (see Methods). Similarly, fractions of cattle, feral pig and water samples positive for a least one non-O157 STEC (B) and monthly rainfall totals are shown for a 10 month period (Jan 2010– Oct 2010) from the same 4 weather sites used in panel A. Letters to the left of the plots indicate significant (P<0.05) correlation between those plots with the same letter designation.

During the 2.5 year sampling period, the incidence of O157 in cattle was significantly higher in the summer months of July through October (P = 0.008, Figure 7A). Although O157 in feral pigs was not seasonal significantly, the monthly incidence of O157 in feral pigs correlated with that of O157 in cattle (r = 0.62, P = 0.032), due primarily to the increased incidence in feral pigs during July, August and October. In contrast, non-O157 STEC incidence in cattle and feral pigs failed to show any significant seasonality (Figure 7B). However, non-O157 STEC from feral pigs correlated with the monthly non-O157 STEC from water (r = 0.70, P = 0.033). Additionally, O157 correlated with non-O157 STEC values in water (r = 0.87, P = 0.001) and both were significantly higher during the months of January, February and March (P = 0.028) with higher monthly rainfall totals (r = 0.61, P = 0.033 and r = 0.80, P = 0.005, respectively). O157 and non-O157 STEC in cattle (r = −0.32, P = 0.36) and in feral pigs did not correlate (r = −0.17, P = 0.66). O157 and non-O157 STEC in other wild animal feces was too low, or sampled too intermittently, to yield significant results of seasonal variation (data not shown). Non-O157 STEC incidence in soil and produce samples was too low also (<0.5%) to yield significant seasonal data.

### Phylogenetic Comparison of O157 STEC Strains by an 11-loci MLVA

**Figure 8:**
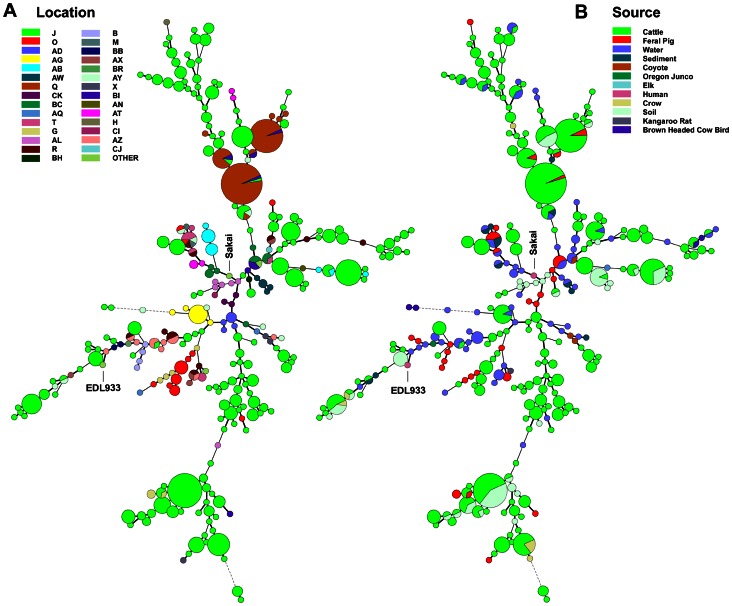
Phylogeny of O157 STEC by 11-loci MLVA. A minimal spanning tree was constructed of 278 MLVA types representing the O157 STEC strains isolated by M3. Node size indicates the relative number of isolates of that type; i.e. the smallest size node represents a single strain of that type. The nodes are color-coded by farm/ranch site code (Panel A) and by sample source (Panel B). Human clinical isolates EDL933 and Sakai are included for comparison only.

One of the goals of this study was to measure point sources of STEC and potential transport processes relevant to produce contamination. Figure 8 illustrates the diversity of O157 STEC strains and the locations and sources associated with the strain MLVA types. The trees in the two panels are identical, but the colored nodes and sectors represent the different farm/ranch codes (Figure 8, Panel A) or sources (Panel B) of the samples yielding O157 STEC. O157 STEC genome strains EDL933 and Sakai have been included as a comparison of MLVA type to the study strains.

The 670 O157 STEC isolates typed by 11-loci MLVA represent 278 different MLVA types. MLVA types 572, 778, 770 and 524 correspond to 47, 33, 31 and 19 isolates, respectively, and were predominately from cows at two locations (locations J and Q, Figure 8). This is seen as largest nodes in the trees, corresponding to multiple O157 STEC positive cattle samples collected on the ranches, usually, on the same day (Panel B, green). This result is consistent with the large number samples tested from multiple ranches and the high incidence of O157 STEC on some ranches (Figure 8, Panel A).

Three large nodes at the top of the Panel A tree represent multiple strains of the same MLVA type isolated predominantly from cattle (Panel B) at location Q, but with a few strains of the same type isolated also from feral pigs at the same or other locations (smaller sectors). There are four smaller nodes representing strains isolated from three or five locations (Panel A) from combinations of cattle, feral pig, water and sediment samples (Panel B). These results suggest routes whereby strains could move by wildlife or water between locations.

### Phylogenetic Comparison of Non-O157 STEC Strains Based on 7-loci MLVA and *ompA* Sequencing Typing

**Figure 9:**
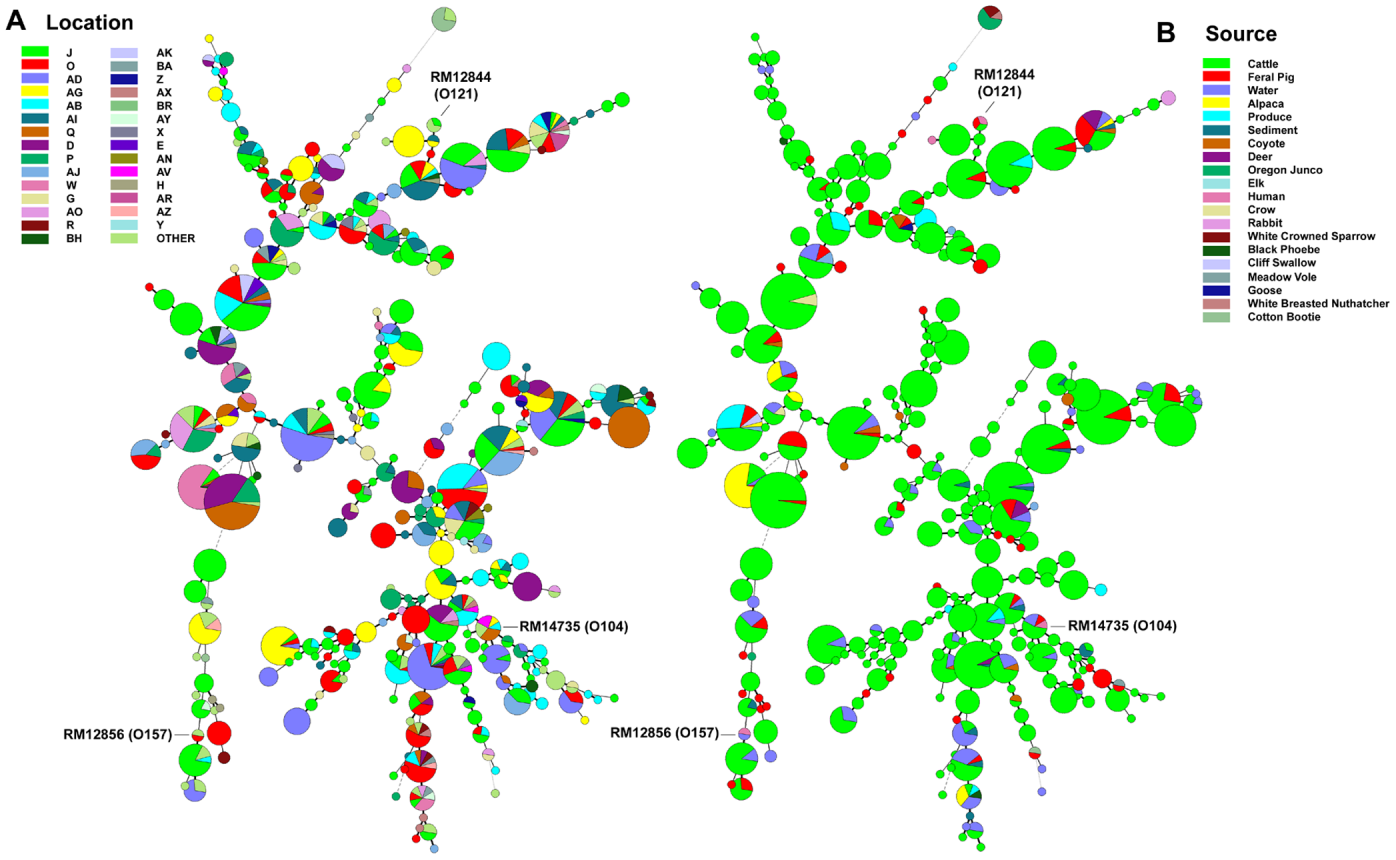
Phylogeny of non-O157 STEC by 7-loci MLVA and *omp*A sequence analysis. A minimal spanning tree was constructed of 286 MLVA/*ompA* types representing the non-O157 STEC strains isolated by M3. Node size indicates the relative number of isolates of that type; i.e. the smallest size node represents a single strain of that type. The nodes are color-coded by farm/ranch site code (Panel A) and by sample source (Panel B). Human clinical isolates RM12844 and RM12856 (OregonPublic Health, 2010) and RM14735 (Germany Fenugreek Outbreak strain, MA Dept. Public Health 2011) are included for comparison only.

Figure 9 illustrates the diversity of non-O157 STEC strains isolated by M3 based on combined MLVA and *omp*A sequence data. Cattle represent the largest number of STEC strains and strain diversity by MLVA-*omp*A. However, multiple nodes are evident with up to 7 different sources represented (e.g. Panel A, top: alpaca, cattle, coyote, deer, feral pig, sediment and water). There are numerous MLVA-*omp*A nodes with both cattle and feral pig STEC represented (Panel A; green and red sectors), suggesting co-mingling of these animals or exposure to similar sources. Similarly, there are numerous MLVA-*omp*A nodes representing matching STEC strains isolated from multiple farm/ranch locations (Panel B). For example, one MLVA-*omp*A type was isolated from 12 different locations (Panel B, top; same node representing 7 sources in Panel A). There are 33 MLVA-*omp*A types that were isolated from at least four different farm/ranch locations. The diameter of some of the nodes indicates multiple strains of that type isolated either on the same day or on different days. Some types are highly related by single or double tandem repeats (i.e. adjacent nodes in the trees), but, overall, the data reflect the diversity and dissemination of STEC in this region.

## Discussion

### Modifications of the Initial STEC Isolation Method

We processed a large and diverse set of environmental samples over a 2.5 year period from 55 ranches, farms and watershed sites in Monterey and San Benito counties of California with sequential versions of our method (M1, M2, M3). An effective method for recovery of STEC strains will depend upon the complexity of the microflora in the sample. To isolate potentially any STEC from a diverse variety of sample types requires a robust method for isolating strains resistant to selection factors and a non-selective approach for isolating potentially important strains that are sensitive to selective methods. Our approach, to add both selective and non-selective methods, was fortuitous considering that some STEC strains were recovered only by non/less-selective methods. We compared methods for isolation of non-O157 STEC by testing a subset of the samples in parallel by the current and modified methods; increases in positive samples were evident. A variety of colonies on NT-RA with distinctive colors were *stx*-positive (Figure 2). Recent publications have reported results consistent with this observation [28], [30]. The use of mSBA has been reported recently as effective in isolation of non-O157 STEC from ground beef and meat samples [20]. Thus, we added this step as the third and final variation of our method (M3). It is important to note that every sample (>13,500 samples) was enriched and tested by O157-IMS beads plated on two selective/indicator media with antibiotics (NT-RA and CT-SMAC) for isolation of O157:H7 strains. Therefore, O157 STEC data can be compared independent of the methods for isolation of non-O157 STEC.

The culture enrichment step is important for isolating STEC. An optimal enrichment medium facilitates rapid growth of any STEC and limits growth of other microflora that can confound identifying STEC. TSB provides an effective and economic medium for efficient enrichment of all STEC strains we have tested. Comparison of TSB with other enrichment media (e.g. Rapid Check and Lactose Broth) supported using TSB (Table 2) and the results obtained were consistent with those reported previously [31], [32]. Dry soil and leafy green samples are stressful environments for enteric bacteria due, probably, to lack of moisture, nutrients and competing microflora, necessitating steps to resuscitate injured cells [33]. Therefore, the use of antibiotics or other selective agents at this critical step may be counter-productive, especially for isolation of a wide range of non-O157 STEC [31], [32], [34]. Indeed, approximately 85% of our non-O157 STEC strains isolated from environmental samples on non-selective media (mSBA and C-O157) were unable to grow on selective media (data not shown).

The results of spiking different environmental sample types with O157:H7, including stressed O157:H7 cells isolated from very dry soil, indicated that detection sensitivity, generally, was below 10 CFU per enrichment sample. These results are similar to the sensitivity reported previously for isolation methods for meat and feces [28], [31], [35]. Increasing the enrichment incubation time did not improve sensitivity, consistent with the work of Ogden et. al. [36]. The only exception to the 10 CFU per enrichment sensitivity was a group of fecal samples that contained large amounts of suspended material that appeared to be associated with poor recovery of IMS beads. We speculate that particles in the fecal sample bind hydrophobically to beads, causing beads to be repelled from the wall of the tube and limiting capture of the beads by the magnet [37].

### Detection of *stx2* Genes in TSB Enrichment Cultures

Our initial non-O157 STEC isolation method (M1) relied on detection of *stx* genes in TSB enrichment culture before plating on C-O157. Many PCR methods have been designed for high throughput detection of *stx* (RT multiplex) [9], [28], [38], [39], [40], [41], [42], [43], [44], [45], [46], [47], [48], [49], [50], [51], [52], but few have been tested for detection of *stx* in primary enrichment cultures [28], [38], [40], [42], [50]. Indeed, none of the reported methods was designed to detect all of the known *stx* types, probably, due to the divergence of the *stx*2 family, in addition to the lack of association of some of the *stx*2 types with clinical illness, outbreaks or even isolation from human feces [53], [54]. For example, the most divergent *stx2* type, *stx*2f, is isolated most frequently from avian species and detected rarely in human feces [55]. Nevertheless, our method was developed to detect and recover as many STEC as possible from agricultural/environmental samples for the purpose of source tracking, regardless of their clinical significance.

### Sensitivity of *stx* Detection by PCR of Enrichments

The sensitivity of the multiplex PCR reaction was evaluated by inoculation of *E. coli* O157 into STEC-negative enrichments of feces, soil, produce and water samples. These enrichments contained complex microflora typical for these sample types. In sample types without any enrichment of the added *E. coli* O157, *stx* was detectable at 3.5×10^6^ CFU/mL. This level of sensitivity was achieved only after eliminating the effect of PCR inhibitors, in soil and feces especially, by filtration and/or using a commercial supplement (EMM, see methods). This sensitivity is less than the sensitivities of 10^3^–10^4^ CFU/mL reported for other PCR methods [28], [38], [40], [42], [50], some with complex enrichments. *E. coli* O157 spiked into our enrichments at 10^3^ CFU/mL were also detectable, but the Ct values were over 30 (data not shown). TSB enrichment broth is non-selective, thus many non-target bacteria are present, sometimes as high as 10^9^ CFU per mL (data not shown). Therefore, the chance of recovering non-O157 STEC isolates on a non-selective medium such as C-O157 by random picking of colonies is low. However, *E. coli* colonies on C-O157 plates were blue >80% of the time (Figure 1, center panel A and Figure 2), thus picking only blue colonies facilitated isolating STEC. Nevertheless, we determined that success in isolation of non-O157 STEC from samples yielding a *stx*-PCR value >Ct of 27 was low, based on experience with thousands of samples. The rate of non-O157 STEC recovery with Ct values of 27 was <10% (Figure 3). As a comparison, pure STEC isolates produced Ct values of 16–18, indicating the STEC cell concentration in a “Ct = 27″ enrichment, theoretically, was less than 0.1% of total cells (a Ct change of 3.3 is roughly equivalent to a 10 fold change in template concentration).

The sensitivity of the *stx* multiplex PCR between enrichments varied. For example, *E. coli* O157 RM1484 added to enrichments of soil and feces often resulted in no amplification of the *stx* genes (Table 3). Enrichments even with >2×10^7^ CFU of *E. coli* O157 RM1484 per mL (10^5^ CFU per PCR reaction) were undetectable for *stx* occasionally. Washing the cells in the enrichments by a centrifugation step prior to boiling failed to eliminate inhibitors (data not shown). Five logs of cells alone in control reactions were sufficient to produce a Ct of 17, indicating the presence of PCR inhibitors in soil and feces interfering with amplification. Although we determined that PCR inhibitors could be decreased or eliminated using a DNA extraction and purification kit from one of several manufacturers, the cost and time commitment necessary were deemed prohibitive for a large study. However, a significant improvement in sensitivity of the *stx* PCR with enrichment broths was achieved by the addition of EMM (Life Tech., ABI), thus facilitating efficient identification of enrichments for subsequent plating on C-O157 (Table 3).

### Culture Bias for Non-O157 STEC Recovery from Different Media

Anti-O157 antibody on commercial magnetic beads may be quite specific for O157 antigen, but the diverse types of non-O157 colonies on NT-RA, including non-O157 *E. coli*, made it evident that other bacteria were bound to the beads (Table 5) [56]. Antibody specificity may decrease in complex enrichment broths due to changes in ionic strength, microflora or other unknown factors. A potential advantage of this non-specificity is that other types of STEC can be isolated from the same samples by different media/methods, thus, providing a multi-pronged strategy for robust isolation of non-O157 STEC in environmental samples. For example, different culture methods appeared to enhance recovery of specific O-types (Figure 6). The “PCR method” recovered O45, O91, O113 and O121 STEC from C-O157 plates more frequently compared to the “IMS method” with NT-RA. In contrast, the IMS method (Figure 6, “NT-Rainbow”) recovered O26, O103, O111 and O145 STEC more frequently than the PCR method. These O-types represent four of the five non-O157 STEC O-types most frequently associated with HUS [57], [58].

### Virulence Gene Profile of STEC Isolated by PCR and IMS Methods

Of the 670 O157 strains isolated by our method, all were typical H7 by PCR (*fliC*_H7_-positive) and positive for the intimin (*eae*) gene. A large percentage of the O157 strains (97–99%) also contained *stx2* and *hlyA*. It is significant that no non-H7 or non-*eae* ^+^ O157 strains were isolated despite the fact that they have been isolated previously from animals, surface water, food and humans throughout the world [59], [60]. It is possible that there is a very low incidence of these atypical O157 in our sampling region or they are not easily recognizable by our methods. This result is consistent with only typical O157:H7, *eae* ^+^ strains isolated in our previous study of the same watershed [21], but is in contrast to H7-negative O157 strains isolated in a similar study in Mexico using a similar IMS procedure [61]The Mexico O157 strains were determined to be *stx*-negative, but positive for *eae*.

The distinct serotype differences in the non-O157 STEC isolated by the IMS and PCR methods stimulated us to compare the virulence gene profiles of the non-O157 STEC isolated by the two methods. The O-types isolated corresponded to the presence of several important virulence genes (e.g. *eae*) detected by the IMS-method more frequently than the PCR-method. Furthermore, the frequency of isolation of *eae*+ strains from IMS-plated bacteria on mSBA was the same as the PCR method (i.e. no IMS), but was approximately 5-fold lower than recovery from IMS beads plated on NT-RA, indicating the difference is not due to IMS alone (data not shown).

These results confirm that STEC strains have differential fitness on these media. This can result from sensitivity to antibiotics in selective media, nutrient preferences or microflora overgrowth. For example, only 20% of our produce isolates (n = 12) were recovered by IMS on NT-RA; however, they were all *eae* ^+^ isolates (Table 6 and Figure 6). Previous reports confirm, however, that intimin-negative STEC can be virulent [62], [63], [64]. Indeed, a recent, large outbreak in Germany associated with fenugreek sprouts was caused by an *eae*-negative O104 STEC that was hypervirulent based on the significantly high level of HUS [17]. This suggests that other attachment factors substitute for intimin. For example, the gene for the autoagglutinating adhesion, *saa* (Figure 6), was present in strains of the O45 and O113 O-types, which were isolated preferentially by our PCR-method [23].

The reason(s) for apparent “culture bias” related to O-types and virulence factors is unclear, but probably involves fitness differences on NT-RA and C-O157 due to ingredients selective for certain STEC or, alternatively, the lack of ingredients beneficial to growth of certain non-O157 STEC. Another factor we considered to explain this “bias” was antibodies on the IMS beads other than anti-O157 that might bind outer surface antigens on STEC. However, a sample of the antibody provided by the manufacturer and tested in SDS-PAGE immunoblotting assays with a set of STEC did not reveal any significant binding to surface molecules other than O157 LPS (data not shown). Although additional parallel experiments will be required to clarify the mechanisms contributing to culture bias, the results emphasize the value of using multiple media and approaches for robust detection and isolation of non-O157 STEC.

### STEC O-types Isolated from Environmental Samples

Despite the fact that our culture method was effective with diverse sample types (e.g. water, livestock and wildlife feces, soil, plants), it is unlikely that public health and regulatory laboratories would adapt them currently, because these labs are focusing their efforts currently on specific non-O157 STEC accounting for >80% of clinical illness [65]. For example, specific IMS beads have been developed commercially for enhancing the efficiency of isolating O26, O103, O111 and O145 strains [31], [66]. We tested these commercial reagents in an experiment with complex enrichments of a group of environmental samples inoculated with specific O-type STEC and did not observe significant improvement in STEC isolation compared to the O157-IMS beads (data not shown). This may be due to the complexity of the microflora and inhibitors present in our non-meat environmental samples. Most of the clinically relevant O-types in our study were isolated with O157-IMS beads plated on NT-RA, however, it remains possible that our strategy lacking O-type-specific IMS analogous to the O157 method decreases the efficiency at recovering some O-types (Figures 4 and 5). O-types exhibited colony morphology differences similar to those noted by Fratamico et. al. with NT-RA [28], but varied more than observed for the “top 6 O-types” ([28]; and Figure 6). A recent report on isolation of the “top 6″ STEC O-types from ground beef [30] uses NT-RA modified to contain approximately 5-fold less tellurite and half the novobiocin of a previous USDA FSIS method, but the addition also of cefixime plus acid treatment of the IMS beads before plating are modifications necessary probably for isolation of relevant STEC strains not recoverable by the USDA FSIS previous method.

### Summary of the Incidence of STEC in Environmental Samples

Our goal for the study described in this report was to develop a robust method for isolating, as efficiently as possible, both O157 and non-O157 STEC strains from multiple sources relevant to outbreaks and recalls in a major leafy greens production ecosystem. The farm and ranch locations are blinded for the results of this preliminary analysis for reasons of confidentiality. A more comprehensive and detailed statistical analysis of STEC incidence, genotyping data and field data (e.g. climate conditions, irrigation and production practices, distance from livestock, cow/calf details, riparian zones, etc.) to determine matches of strains (based on MLVA for both O157 and non-O157 STEC and PFGE for O157) to outbreaks (predominantly O157) and patterns of STEC incidence and movement related to agricultural practices, will be presented in a future report (manuscript in preparation).

Characterization of non-O157 STEC strains by O-type ELISA and PCR revealed evidence of culture bias, but also the advantage of using different media and strategies for efficient isolation of non-O157 STEC from complex environmental samples representing diverse hosts, matrices and microflora. We are unaware of any other reports that have surveyed by a comparable strategy a broad range of STEC from multiple types of complex environmental samples rather than spiking studies with a limited set and less diverse reference strains. A disadvantage of our survey with modified methods used at different periods is that comparing results is not possible with high statistical significance. However, the results provide evidence of improvements in sensitivity for recovering STEC and differences in the types of strains cultured best by each method. Isolation of diverse types of STEC strains is critical for microbial source tracking, outbreak epidemiology and incidence of virulent STEC in the environment and food [67].

A summary of the results obtained for all three methods shown in 1 (“M1+M2+M3”) indicates that domestic ruminants are often positive for STEC (6.6% O157, 36% non-O157). Water sources ranged from 8.2% of samples positive on produce farms to 17.8% on ranches. The incidence of STEC in wildlife was similar regardless of method (Table 4; 6.8–7.8%); *stx*, *subA*, *eae* and *ehxA* genes were present at levels similar to livestock (Table 6). Considering the large number of wildlife samples tested (3,202) from a variety of species (e.g. deer, elk, feral pig, small mammals, birds), an incidence of STEC of approximately 7% overall points to other means of transport from ranches and/or watersheds to produce on farms.

The 110 samples positive for non-O157 STEC only with mSBA (Figure 5, “B”) are consistent with higher incidence of STEC with M3 (Figure 4 and Table 6). The incidences of non-O157 STEC for each method (M1, M2, M3) are not easily comparable because of different samples analyzed at sequential periods, but comparison of results obtained with our final STEC method (M3) for the last period of the survey (Jan to Oct, 2010) indicates potential seasonality of O157 STEC incidence in cattle (Figure 7, panel A: Jul-Oct). These results are consistent with trends in seasonality described from a survey of cattle at beef processing facilities in 2001–2002 and other studies of STEC [68], [69], [70], [71], [72]. It is intriguing that the incidence of O157 STEC in feral pigs during summer and fall months correlated generally with incidence in cattle (Figure 7, panel A); feral pig activity was speculated as a risk in the 2006 O157 STEC outbreak associated with baby spinach, which started in August of that year [73]. In contrast, non-O157 STEC incidence in cattle was stable overall relative to sampling month (with an exception of July), ranging from approximately 30 to 65%; correlation to incidence in feral pigs was minimal (Figure 7, panel B).

The average O157 incidence in cattle reflects the incidence on 6 of the 10 ranches included in the study with two of the ranches (J and O) accounting for 73.8% and 16.7% of the positives, respectively (data not shown). The ranch having the highest O157 STEC incidence in cattle was sampled on 14 different dates between Jul-2008 to Oct-2010; Jul-08, Jul-09 and Oct-10 resulted in 38%, 35% and 39% of the samples tested being positive. The second ranch was positive on only 1 of 10 sampling dates, but 90% of the samples were positive on that date. In contrast, multiple cow samples on all 10 ranches were positive for non-O157 STEC on at least one date, and usually multiple dates, at incidence levels ranging between 15–80%, but lacked any seasonal correlation (data not shown). These results confirm that most ranches in this region have low incidence of O157 STEC, but a relatively stable and frequent incidence of O157 STEC was detected on one ranch and a transient high incidence was detected on another ranch. These results will be evaluated with field data acquired during the study and presented in a future report.

The overall incidence of both O157 and non-O157 STEC in water samples was highest during spring months corresponding to the highest rainfall (Figure 7, Jan-Mar), but with minor O157 spikes in Aug and Oct, is consistent with the incidence we reported previously for *E. coli* O157 in the Salinas Valley watershed in 2005–2006 [21]. We noted a positive correlation between non-O157 STEC incidence in feral pigs and watershed samples, possibly indicating an impact of fecal shedding on surface water contamination (Figure 7). In contrast, O157 and non-O157 STEC incidence levels in either cattle or feral pig feces indicated that animals positive for O157 do not correlate with the possibility that they carry other STEC serotypes.

### Source Tracking of O157 STEC Strains by 11-loci MLVA

The O157 STEC strains isolated are diverse corresponding to a large number of different nodes seen in Figure 8. Many of the small nodes adjacent in the tree represent single tandem repeat differences in a single locus. Frequently, this difference is within the Vhec1 locus, which is the most variable locus in our MLVA method [21], [24]. The numerous single color nodes reflect strains isolated from only one location (Panel A), and a large number of diverse strains from one location (Panel A, Location J) and from one source (Panel B, cattle).

A comparison of O157 STEC MLVA data from this study to our larger, internal O157 STEC MLVA database consisting of >1100 different MLVA types revealed matches to human strains associated with at least four outbreaks reported by CDC (see [74] for other CDC MLVA and PFGE O157 outbreak-related comparisons). We became aware of these links to outbreaks because of *XbaI* and *BlnI* profiles submitted to CDC PulseNet [75] matching profiles of clinical strains for at least four different previous and/or ongoing outbreaks. We obtained clinical strains associated with three outbreaks (CDC outbreak designations 0609mlEXH-2, 0704mEXH-1c, 0809MIEXH-1c) and they either were identical, or highly related, by 11-loci MLVA to strains collected in this study (data not shown). Matching strains corresponding to three of the outbreaks associated with leafy greens, or suspected leafy greens in 2006, 2007 and 2008 were isolated from a bird, cows, and a watershed and a coyote, respectively. Four of the outbreaks occurred at periods consistent with harvests from the survey region suggesting that the environmental strains are spatially and/or temporally relevant to these outbreaks.

### Source Tracking of Non-O157 STEC Strains by 7-loci MLVA-ompA Typing

All of the isolates confirmed as non-O157 STEC were typed by 7-loci MLVA-*omp*A method for the purpose of identifying potential point sources and patterns of movement. The non-O157 STEC strains are diverse phylogenetically, and some were present at multiple locations (Figure 9, Panel A) and sources (Panel B). The largest nodes shown in Figure 9 (i.e., multiple strains of that MLVA-*omp*A type) reveal that many of the strains isolated from >5 locations (Panel A) were isolated from cattle ranches (Panel B, green sectors). These results are consistent with previous studies of the incidence of non-O157 STEC in cattle and beef products [20], [76]. Also, at least 10 of the large nodes shown in Panel B represent strains isolated from cattle on multiple ranches and feral pigs on at least one of the ranches (Figure 9, Panel B, green and red sectors). Although it is not possible from these data to determine how cattle and feral pigs were first exposed to the non-O157 STEC strains, it is logical to assume from these results that co-mingling of cattle and other wildlife occurs, leading to the spread of the microflora (Panel B) [73]. Many of the wildlife species positive for O157 (bird, coyote, elk, feral pig) or non-O157 STEC (bird, coyote, deer, elk, feral pig) roam long distances, emphasizing the potential transport of STEC to produce.

Other factors relevant to risk assessments of STEC in this environment are the quantity (i.e. concentration in sample, amount of sample) and virulence of these STEC strains (Figure 6). We did not measure the concentration of STEC in each sample, because, previous experience revealed that the concentration usually is well below the level of detection by direct plating. Occasionally, we did detect very high-shedding cows (>10^6^ cfu/g feces) and a couple relatively high-shedding feral pigs (>10^4^ cfu/g feces) by direct plating of samples we suspected as being high concentrations based on almost pure O157 STEC colonies from enrichment broths plated on NT-RA. Animals shedding high levels of pathogenic strains are relevant epidemiologically, because they are responsible probably for the predominance of some strains in the environment (Figures 8 and 9). Predominant strains that are highly virulent also would be probable candidates for links to foodborne outbreaks [77]. *E. coli* O157:H7 strains linked to outbreaks associated with bagged leafy vegetables are part of a phylogenetically distinct group (“clade 8″) causing severe illness [78]. Therefore, high-shedder animals and STEC strain fitness and/or virulence ([79] and Figure 6) are important factors in the epidemiology of STEC in food production environments and foodborne outbreaks.

We developed a robust method for isolating STEC during a large survey of environmental samples. A comparison of the STEC incidence results by the three versions of the method revealed evidence of culture bias and the value of multiple culture strategies. Characterization of different types of STEC provides data that are valuable for future studies of the epidemiology of STEC in food production environments and the biology of STEC fitness and virulence. A robust method will be useful especially for investigation of recalls or outbreaks traced to this important agricultural region.

## Supporting Information

Table S1 **Multiplex PCR amplification of selected non-O157 STEC strains using an STEC collection received from** ***E. coli*** **Reference Center (ECRC) at Penn State University (PSU).**
(DOCX)

## References

[1] Rangel JM, Sparling PH, Crowe C, Griffin PM, Swerdlow DL (2005) Epidemiology of *Escherichia coli* O157:H7 outbreaks, United States, 1982–2002. Emerging Infectious Diseases 11: 603–609. 15829201 10.3201/eid1104.040739 PMC3320345

[2] Jelacic JK, Damrow T, Chen GS, Jelacic S, Bielaszewska M, et al (2003) Shiga toxin-producing Escherichia coli in Montana: bacterial genotypes and clinical profiles. J Infect Dis 188: 719–729. 12934188 10.1086/376999

[3] Manning SD, Madera RT, Schneider W, Dietrich SE, Khalife W, et al (2007) Surveillance for Shiga toxin-producing Escherichia coli, Michigan, 2001–2005. Emerg Infect Dis 13: 318–321. 17479902 10.3201/eid1302.060813 PMC2725841

[4] Anonymous (2007) Centers for Disease Control and Prevention. Laboratory-confirmed non-O157 Shiga toxin-producing *Escherichia coli*–Connecticut, 2000–2005. Morbidity and Mortality Weekly Reporter 56: 29–31. 17230143

[5] Hedican EB, Medus C, Besser JM, Juni BA, Koziol B, et al (2009) Characteristics of O157 versus non-O157 Shiga toxin-producing Escherichia coli infections in Minnesota, 2000–2006. Clin Infect Dis 49: 358–364. 19548834 10.1086/600302

[6] Beutin L, Krause G, Zimmermann S, Kaulfuss S, Gleier K (2004) Characterization of Shiga toxin-producing Escherichia coli strains isolated from human patients in Germany over a 3-year period. J Clin Microbiol 42: 1099–1108. 15004060 10.1128/JCM.42.3.1099-1108.2004 PMC356890

[7] Brooks JT, Sowers EG, Wells JG, Greene KD, Griffin PM, et al (2005) Non-O157 Shiga toxin–producing *Escherichia coli* infections in the United States, 1983–2002. The Journal of Infectious Diseases 192: 1422–1429. 16170761 10.1086/466536

[8] Willford J, Mills K, Goodridge LD (2009) Evaluation of three commercially available enzyme-linked immunosorbent assay kits for detection of Shiga toxin. Journal of Food Protection 72: 741–747. 19435221 10.4315/0362-028x-72.4.741

[9] Grys TE, Sloan LM, Rosenblatt JE, Patel R (2009) Rapid and sensitive detection of shiga toxin-producing *Escherichia coli* from nonenriched stool specimens by real-time PCR in comparison to enzyme immunoassay and culture. Journal of Clinical Microbiology 47: 2008–2012. 19439539 10.1128/JCM.02013-08 PMC2708480

[10] Zheng J, Cui S, Teel LD, Zhao S, Singh R, et al (2008) Identification and characterization of Shiga toxin type 2 variants in *Escherichia coli* isolates from animals, food, and humans. Applied and Environmental Microbiology 74: 5645–5652. 18658282 10.1128/AEM.00503-08 PMC2547040

[11] Allerberger F (2009) Incidence and microbiology of salad-borne disease. CAB Reviews: Perspectives in Agriculture, Veterinary Science, Nutrition and Natural Resources 4: 1–13. 10.1079/pavsnnr202116039 PMC8580373 34765015

[12] Ingham SC, Fanslau MA, Engel RA, Zhu J, Breuer JR, et al (2005) Evaluation of fertilization-to-planting and fertilization-to-harvest intervals for safe use of noncomposted bovine manure in Wisconsin vegetable production. Journal of Food Protection 68: 1134–1142. 15954698 10.4315/0362-028x-68.6.1134

[13] Islam M, Doyle MP, Phatak SC, Millner P, Jiang X (2005) Survival of *Escherichia coli* O157:H7 in soil and on carrots and onions grown in fields treated with contaminated manure composts or irrigation water. Food Microbiology 22: 63–70.

[14] Johannessen GS, Heier BT, Rørvik LM, Bengtsson GB, Bredholt S, et al (2005) Potential uptake of *Escherichia coli* O157:H7 from organic manure into crisphead lettuce. Applied and Environmental Microbiology 71: 2221–2225. 15870303 10.1128/AEM.71.5.2221-2225.2005 PMC1087526

[15] Solomon EB, Yaron S, Matthews KR (2002) Transmission of *Escherichia coli* O157:H7 from contaminated manure and irrigation water to lettuce plant tissue and its subsequent internalization. Applied and Environmental Microbiology 68: 397–400. 11772650 10.1128/AEM.68.1.397-400.2002 PMC126537

[16] Anonymous-FDA (2010) Federal and state officials confirm link between bagged Romaine lettuce and *E. coli* O145 illness outbreak. In: FDA, editor. FDA.

[17] Frank C, Faber M, Askar M, Bernard H, Fruth A, et al.. (2011) Large and ongoing outbreak of haemolytic uraemic syndrome, Germany, May 2011. Euro Surveill: Euro Surveill. 1–5. 21632020

[18] Bielaszewska M, Mellmann A, Zhang W, Köck R, Fruth A, et al (2011) Characterisation of the *Escherichia coli* strain associated with an outbreak of haemolytic uraemic syndrome in Germany, 2011: a microbiological study. The Lancet Infectious Diseases 11: 671–676. 21703928 10.1016/S1473-3099(11)70165-7

[19] Barrett TJ, Blake PA, Morris GK (1980) Use of Moore swabs for isolating *Vibrio cholerae* from sewage. Journal of Clinical Microbiology 11: 385–388. 6989857 10.1128/jcm.11.4.385-388.1980 PMC273409

[20] Bosilevac JM, Koohmaraie M (2011) Prevalence and characterization of non-O157 Shiga toxin-producing *Escherichia coli* isolates from commercial ground beef in the United States. Applied and Environmental Microbiology 77: 2103–2112. 21257806 10.1128/AEM.02833-10 PMC3067332

[21] Cooley M, Carychao D, Crawford-Miksza L, Jay MT, Myers C, et al (2007) Incidence and tracking of *Escherichia coli* O157:H7 in a major produce production region in California. PLoS ONE 2: e1159. 18174909 10.1371/journal.pone.0001159 PMC2174234

[22] Rivera-Betancourt M, Keen JE (2000) Murine monoclonal antibodies specific for lipopolysaccharide of *Escherichia coli* O26 and O111. Applied and Environmental Microbiology 66: 4124–4127. 10966439 10.1128/aem.66.9.4124-4127.2000 PMC92269

[23] Quinones B, Swimley MS, Narm KE, Patel RN, Cooley MB, et al (2012) O-antigen and virulence profiling of Shiga toxin-producing *Escherichia coli* by a rapid and cost-effective dna microarray colorimetric method. Front in Cell Infect Microbiol 2: 61. 22919652 10.3389/fcimb.2012.00061 PMC3417394

[24] Cooley MB, Carychao D, Nguyen K, Whitehand L, Mandrell R (2010) Effects of environmental stress on stability of tandem repeats in *Escherichia coli* O157:H7. Appl Environ Microbiol 76: 3398–3400. 20348301 10.1128/AEM.02373-09 PMC2869130

[25] Lindstedt BA, Brandal LT, Aas L, Vardund T, Kapperud G (2007) Study of polymorphic variable-number of tandem repeats loci in the ECOR collection and in a set of pathogenic *Escherichia coli* and Shigella isolates for use in a genotyping assay. Journal of Microbiological Methods 69: 197–205. 17291612 10.1016/j.mimet.2007.01.001

[26] Smith SGJ, Mahon V, Lambert MA, Fagan RP (2007) A molecular Swiss army knife: OmpA structure, function and expression. FEMS Microbiology Letters 273: 1–11. 17559395 10.1111/j.1574-6968.2007.00778.x

[27] Garthright WE, Blodgett RJ (2003) FDA's preferred MPN methods for standard, large or unusual tests, with a spreadsheet. Food Microbiology 20: 439–445.

[28] Fratamico P, Bagi LK, Cray WC Jr, Narang N, Yan X, et al (2011) Detection by multiplex real-time polymerase chain reaction assays and isolation of Shiga toxin–producing *Escherichia coli* serogroups O26, O45, O103, O111, O121, and O145 in ground beef. Foodborne Pathogens and Disease 8: 601–607. 21214490 10.1089/fpd.2010.0773

[29] Sugiyama K, Inoue K, Sakazaki R (2001) Mitomycin-supplemented washed blood agar for the isolation of Shiga toxin-producing Escherichia coli other than O157:H7. Lett Appl Microbiol 33: 193–195. 11555202 10.1046/j.1472-765x.2001.00974.x

[30] Tillman GE, Wasilenko JL, Simmons M, Lauze TA, Minicozzi J, et al (2012) Isolation of Shiga toxin-producing *Escherichia coli* serogroups O26, O45, O103, O111, O121, and O145 from ground beef using modified rainbow agar and post-immunomagnetic separation acid treatment. Journal of Food Protection 75: 1548–1554. 22947460 10.4315/0362-028X.JFP-12-110

[31] Verstraete K, De Zutter L, Messens W, Herman L, Heyndrickx M, et al (2010) Effect of the enrichment time and immunomagnetic separation on the detection of Shiga toxin-producing *Escherichia coli* O26, O103, O111, O145 and sorbitol positive O157 from artificially inoculated cattle faeces. Veterinary Microbiology 145: 106–112. 20378282 10.1016/j.vetmic.2010.03.004

[32] Tzschoppe M, Martin A, Beutin L (2011) A rapid procedure for the detection and isolation of enterohaemorrhagic Escherichia coli (EHEC) serogroup O26, O103, O111, O118, O121, O145 and O157 strains and the aggregative EHEC O104:H4 strain from ready-to-eat vegetables. International Journal of Food Microbiology 152: 19–30. 22071287 10.1016/j.ijfoodmicro.2011.10.009

[33] Stephens PJ, Joynson JA (1998) Direct inoculation into media containing bile salts and antibiotics is unsuitable for the detection of acid/salt stressed *Escherichia coli* O157:H7. Letters in Applied Microbiology 27: 147–151. 9750318 10.1046/j.1472-765x.1998.t01-1-00415.x

[34] Kanki M, Seto K, Harada T, Yonogi S, Kumeda Y (2011) Comparison of four enrichment broths for the detection of non-O157 Shiga-toxin-producing Escherichia coli O91, O103, O111, O119, O121, O145 and O165 from pure culture and food samples. Letters in Applied Microbiology 53: 167–173. 21585404 10.1111/j.1472-765X.2011.03085.x

[35] Fedio WM, Jinneman KC, Yoshitomi KJ, Zapata R, Wendakoon CN, et al (2011) Detection of E. coli O157:H7 in raw ground beef by Pathatrix™ immunomagnetic-separation, real-time PCR and cultural methods. International Journal of Food Microbiology 148: 87–92. 21641670 10.1016/j.ijfoodmicro.2011.05.005

[36] Ogden ID, Hepburn NF, MacRae M (2001) The optimization of isolation media used in immunomagnetic separation methods for the detection of *Escherichia coli* O157 in foods. Journal of Applied Microbiology 91: 373–379. 11473603 10.1046/j.1365-2672.2001.01397.x

[37] Parham N, Spencer J, Taylor D, Ternent H, Innocent G, et al (2003) An adapted immunomagnetic cell separation method for use in quantification of *Escherichia coli* O157:H7 from bovine faeces. Journal of Microbiological Methods 53: 1–9. 12609717 10.1016/s0167-7012(02)00206-3

[38] Auvray F, Lecureuil C, Dilasser F, Taché J, Derzelle S (2009) Development of a real-time PCR assay with an internal amplification control for the screening of Shiga toxin-producing *Escherichia coli* in foods. Letters in Applied Microbiology 48: 554–559. 19220740 10.1111/j.1472-765X.2009.02561.x

[39] Bélanger SD, Boissinot M, Ménard C, Picard FJ, Bergeron MG (2002) Rapid detection of Shiga toxin-producing bacteria in feces by multiplex PCR with molecular beacons on the Smart Cycler. Journal of Clinical Microbiology 40: 1436–1440. 11923369 10.1128/JCM.40.4.1436-1440.2002 PMC140333

[40] Chassagne L, Pradel N, Robin F, Livrelli V, Bonnet R, et al (2009) Detection of *stx*1, *stx*2, and *eae* genes of enterohemorrhagic *Escherichia coli* using SYBR Green in a real-time polymerase chain reaction. Diagnostic Microbiology and Infectious Disease 64: 98–101. 19362260 10.1016/j.diagmicrobio.2009.01.031

[41] Guion CE, Ochoa TJ, Walker CM, Barletta F, Cleary TG (2008) Detection of diarrheagenic *Escherichia coli* by use of melting-curve analysis and real-time multiplex PCR. Journal of Clinical Microbiology 46: 1752–1757. 18322059 10.1128/JCM.02341-07 PMC2395066

[42] Ibekwe AM, Watt PM, Grieve CM, Sharma VK, Lyons SR (2002) Multiplex fluorogenic real-time PCR for detection and quantification of *Escherichia coli* O157:H7 in dairy wastewater wetlands. Applied and Environmental Microbiology 68: 4853–4862. 12324331 10.1128/AEM.68.10.4853-4862.2002 PMC126415

[43] Jinneman KC, Yoshitomi KJ, Weagant SD (2003) Multiplex real-time PCR method to identify Shiga toxin genes *stx*1 and *stx*2 and *Escherichia coli* O157:H7/H- serotype. Applied and Environmental Microbiology 69: 6327–6333. 14532101 10.1128/AEM.69.10.6327-6333.2003 PMC201207

[44] Nielsen EM, Andersen MT (2003) Detection and characterization of verocytotoxin-producing *Escherichia coli* by automated 5′ nuclease PCR assay. Journal of Clinical Microbiology 41: 2884–2893. 12843017 10.1128/JCM.41.7.2884-2893.2003 PMC165313

[45] Pavlovic M, Huber I, Skala H, Konrad R, Schmidt H, et al (2010) Development of a multiplex real-time polymerase chain reaction for simultaneous detection of enterohemorrhagic *Escherichia coli* and enteropathogenic *Escherichia coli* strains. Foodborne Pathogens and Disease 7: 801–808. 20156086 10.1089/fpd.2009.0457

[46] Perelle S, Dilasser F, Grout J, Fach P (2004) Detection by 5′-nuclease PCR of Shiga-toxin producing *Escherichia coli* O26, O55, O91, O103, O111, O113, O145 and O157:H7, associated with the world's most frequent clinical cases. Molecular and cellular probes 18: 185–192. 15135453 10.1016/j.mcp.2003.12.004

[47] Reischl U, Youssef MT, Kilwinski J, Lehn N, Zhang WL, et al (2002) Real-time fluorescence PCR assays for detection and characterization of Shiga toxin, intimin, and enterohemolysin genes from Shiga toxin-producing *Escherichia coli* . Journal of Clinical Microbiology 40: 2555–2565. 12089277 10.1128/JCM.40.7.2555-2565.2002 PMC120605

[48] Schuurman T, Roovers A, van der Zwaluw WK, van Zwet AA, Sabbe LJM, et al (2007) Evaluation of 5′-nuclease and hybridization probe assays for the detection of shiga toxin-producing *Escherichia coli* in human stools. Journal of Microbiological Methods 70: 406–415. 17614150 10.1016/j.mimet.2007.05.016

[49] Sekse C, Solberg A, Petersen A, Rudi K, Wasteson Y (2005) Detection and quantification of Shiga toxin-encoding genes in sheep faeces by real-time PCR. Molecular and cellular probes 19: 363–370. 16150568 10.1016/j.mcp.2005.06.006

[50] Sharma VK, Dean-Nystrom EA (2003) Detection of enterohemorrhagic *Escherichia coli* O157:H7 by using a multiplex real-time PCR assay for genes encoding intimin and Shiga toxins. Veterinary Microbiology 93: 247–260. 12695048 10.1016/s0378-1135(03)00039-7

[51] Yang JR, Wu FT, Tsai JL, Mu JJ, Lin LF, et al (2007) Comparison between O serotyping method and multiplex real-time PCR to identify diarrheagenic *Escherichia coli* in Taiwan. Journal of Clinical Microbiology 45: 3620–3625. 17728475 10.1128/JCM.00596-07 PMC2168529

[52] Yoshitomi KJ, Jinneman KC, Weagant SD (2006) Detection of Shiga toxin genes *stx*1, *stx*2, and the +93 *uid*A mutation of *E. coli* O157:H7/H-using SYBR® Green I in a real-time multiplex PCR. Molecular and cellular probes 20: 31–41. 16271448 10.1016/j.mcp.2005.09.002

[53] Friedrich AW, Bielaszewska M, Zhang WL, Pulz M, Kuczius T, et al (2002) *Escherichia coli* harboring Shiga toxin 2 gene variants: frequency and association with clinical symptoms. Journal of Infectious Diseases 185: 74–84. 11756984 10.1086/338115

[54] Feng PC, Jinneman K, Scheutz F, Monday SR (2011) Specificity of PCR and serological assays in the detection of Escherichia coli Shiga toxin subtypes. Applied and Environmental Microbiology 77: 6699–6702. 21803918 10.1128/AEM.00370-11 PMC3187163

[55] Schmidt H, Scheef J, Morabito S, Caprioli A, Wieler LH, et al (2000) A new Shiga toxin 2 variant (Stx2f) from *Escherichia coli* isolated from pigeons. Applied and Environmental Microbiology 66: 1205–1208. 10698793 10.1128/aem.66.3.1205-1208.2000 PMC91964

[56] Tomoyasu T (1998) Improvement of the immunomagnetic separation method selective for *Escherichia coli* O157 strains. Applied and Environmental Microbiology 64: 376–382. 9435093 10.1128/aem.64.1.376-382.1998 PMC124723

[57] Karmali MA, Mascarenhas M, Shen S, Ziebell K, Johnson S, et al (2003) Association of genomic O island 122 of *Escherichia coli* EDL 933 with verocytotoxin-producing *Escherichia coli* seropathotypes that are linked to epidemic and/or serious disease. Journal of Clinical Microbiology 41: 4930–4940. 14605120 10.1128/JCM.41.11.4930-4940.2003 PMC262514

[58] Bielaszewska M, Karch H (2000) Non-O157:H7 Shiga toxin (verocytotoxin)-producing *Escherichia coli* strains: epidemiological significance and microbiological diagnosis. World J of Microbiol Biotech 16: 711–718.

[59] Tóth I, Schmidt H, Kardos G, Lancz Z, Creuzburg K, et al (2009) Virulence genes and molecular typing of different groups of Escherichia coli O157 strains in cattle. Applied and Environmental Microbiology 75: 6282–6291. 19684174 10.1128/AEM.00873-09 PMC2753057

[60] Feng PCH, Keys C, Lacher D, Monday SR, Shelton D, et al (2010) Prevalence, characterization and clonal analysis of Escherichia coli O157: Non-H7 serotypes that carry eae alleles. FEMS Microbiology Letters 308: 62–67. 20487015 10.1111/j.1574-6968.2010.01990.x

[61] Amézquita-López BA, Quiñones B, Cooley MB, León-Félix J, Castro-del Campo N, et al.. (2012) Genotypic Analyses of Shiga Toxin-Producing Escherichia coli O157 and Non-O157 Recovered from Feces of Domestic Animals on Rural Farms in Mexico. PLoS ONE 7. 10.1371/journal.pone.0051565 PMC3519732 23251577

[62] Toma C, Espinosa EM, Song T, Miliwebsky E, Chinen I, et al (2004) Distribution of putative adhesins in different seropathotypes of Shiga toxin-producing *Escherichia coli* . Journal of Clinical Microbiology 42: 4937–4946. 15528677 10.1128/JCM.42.11.4937-4946.2004 PMC525252

[63] Galli L, Miliwebsky E, Irino K, Leotta G, Rivas M (2010) Virulence profile comparison between LEE-negative Shiga toxin-producing *Escherichia coli* (STEC) strains isolated from cattle and humans. Veterinary Microbiology 143: 307–313. 20022185 10.1016/j.vetmic.2009.11.028

[64] Paton AW, Woodrow MC, Doyle RM, Lanser JA, Paton JC (1999) Molecular characterization of a Shiga toxigenic *Escherichia coli* O113:H21 strain lacking *eae* responsible for a cluster of cases of hemolytic uremic syndrome. Journal of Clinical Microbiology 37: 3357–3361. 10488206 10.1128/jcm.37.10.3357-3361.1999 PMC85566

[65] Johnson KE, Thorpe CM, Sears CL (2006) The emerging clinical importance of non-O157 shiga toxin-producing *Escherichia coli* . Clinical Infectious Diseases 43: 1587–1595. 17109294 10.1086/509573

[66] Bonardi S, Foni E, Chiapponi C, Salsi A, Brindani F (2007) Detection of verocytotoxin-producing *Escherichia coli* serogroups O157 and O26 in the cecal content and lymphatic tissue of cattle at slaughter in Italy. Journal of Food Protection 70: 1493–1497. 17612082 10.4315/0362-028x-70.6.1493

[67] Mandrell RE (2011) Tracing pathogens in fruit and vegetable production chains. In: Brul S, Fratamico PM, McMeekin T, editors. Tracing Pathogens in the Food Chain. Cambridge, UK: Woodhead Publishing Ltd. 548–595.

[68] Barkocy-Gallagher GA, Arthur TM, Rivera-Betancourt M, Nou X, Shackelford SD, et al (2003) Seasonal prevalence of Shiga toxin-producing *Escherichia coli*, including O157:H7 and non-O157 serotypes, and Salmonella in commercial beef processing plants. Journal of Food Protection 66: 1978–1986. 14627272 10.4315/0362-028x-66.11.1978

[69] Arthur TM, Keen JE, Bosilevac JM, Brichta-Harhay DM, Kalchayanand N, et al (2009) Longitudinal study of *Escherichia coli* O157:H7 in a beef cattle feedlot and role of high-level shedders in hide contamination. Applied and Environmental Microbiology 75: 6515–6523. 19684164 10.1128/AEM.00081-09 PMC2765151

[70] Bonardi S, Maggi E, Bottarelli A, Pacciarini ML, Ansuini A, et al (1999) Isolation of Verocytotoxin-producing *Escherichia coli* O157:H7 from cattle at slaughter in Italy. Veterinary Microbiology 67: 203–211. 10418874 10.1016/s0378-1135(99)00039-5

[71] Synge BA, Chase-Topping ME, Hopkins GF, McKendrick IJ, Thomson-Carter F, et al (2003) Factors influencing the shedding of verocytotoxin-producing *Escherichia coli* O157 by beef suckler cows. Epidemiology and Infection 130: 301–312. 12729199 10.1017/s0950268802008208 PMC2869966

[72] Ogden ID, MacRae M, Strachan NJC (2004) Is the prevalence and shedding concentrations of *E. coli* O157 in beef cattle in Scotland seasonal? FEMS Microbiology Letters 233: 297–300. 15063499 10.1016/j.femsle.2004.02.021

[73] Jay MT, Cooley M, Carychao D, Wiscomb GW, Sweitzer RA, et al (2007) *Escherichia coli* O157:H7 in feral swine near spinach fields and cattle, central California coast. Emerg Infect Dis 13: 1908–1911. 18258044 10.3201/eid1312.070763 PMC2876768

[74] Hyytia-Trees E, Smole SC, Fields PA, Swaminathan B, Ribot EM (2006) Second generation subtyping: a proposed PulseNet protocol for multiple-locus variable-number tandem repeat analysis of Shiga toxin-producing *Escherichia coli* O157 (STEC O157). Foodborne Pathog Dis 3: 118–131. 16602987 10.1089/fpd.2006.3.118

[75] Ribot EM, Fair MA, Gautom R, Cameron DN, Hunter SB, et al (2006) Standardization of pulsed-field gel electrophoresis protocols for the subtyping of *Escherichia coli* O157:H7, *Salmonella*, and *Shigella* for PulseNet. Foodborne Pathog Dis 3: 59–67. 16602980 10.1089/fpd.2006.3.59

[76] Hussein HS, Bollinger LM (2005) Prevalence of Shiga toxin-producing *Escherichia coli* in beef cattle. J Food Prot 68: 2224–2241. 16245735 10.4315/0362-028x-68.10.2224

[77] Matthews L, Low JC, Gally DL, Pearce MC, Mellor DJ, et al (2006) Heterogeneous shedding of *Escherichia coli* O157 in cattle and its implications for control. Proc Natl Acad Sci U S A 103: 547–552. 16407143 10.1073/pnas.0503776103 PMC1325964

[78] Manning SD, Motiwala AS, Springman AC, Qi W, Lacher DW, et al (2008) Variation in virulence among clades of *Escherichia coli* O157:H7 associated with disease outbreaks. Proc Natl Acad Sci U S A 105: 4868–4873. 18332430 10.1073/pnas.0710834105 PMC2290780

[79] Teunis P, Takumi K, Shinagawa K (2004) Dose response for infection by *Escherichia coli* O157:H7 from outbreak data. Risk Anal 24: 401–407. 15078310 10.1111/j.0272-4332.2004.00441.x

