# Supplementary material for: Development of a Robust Method for Isolation of Shiga Toxin-Positive Escherichia coli (STEC) from Fecal, Plant, Soil and Water Samples from a Leafy Greens Production Region in California
Source: PLoS One. 2013 Jun 6;8(6):e65716. doi: 10.1371/journal.pone.0065716 (PMC3675059; doi:10.1371/journal.pone.0065716)
Supplement: Table S1 — Multiplex PCR amplification of selected non-O157 STEC strains using an STEC collection received from E. coli Reference Center (ECRC) at Penn State University (PSU). (DOCX) [file pone.0065716.s001.docx]

**Table S1**. Multiplex PCR amplification of selected non-O157 STEC strains

|  |  |  |  |  | *stx* multiplex PCR results^d^ | | | |
| --- | --- | --- | --- | --- | --- | --- | --- | --- |
| Strain | O-type^a^ | ECRC^b^ | Source | Genotype^c^ | stx1 | stx2f | stx2abc | stx2ex |
| RM6919 | 26 | 99.0723 | cattle | stx1 | + | - | - | - |
| RM6920 | 26 | 99.0724 | cattle | stx1 | + | - | - | - |
| RM6921 | 26 | 99.0849 | human | stx1 | + | - | - | - |
| RM6922 | 26 | 99.0850 | human | stx1a,stx1c | + | - | - | - |
| RM6923 | 26 | 99.0869 | human | stx1a,stx1c | + | - | - | - |
| RM6924 | 26 | 99.1761 | human | stx1 | + | - | - | - |
| RM6925 | 26 | 99.1773 | human | stx1 | + | - | - | - |
| RM6926 | 26 | 05.2217 | human | stx1 | + | - | - | - |
| RM6927 | 26 | 06.1592 | ? | stx1, stx2 | + | - | + | + |
| RM6928 | 28 | 07.1641 | ? | stx2, stx2d | - | - | + | + |
| RM6932 | 76 | 01.2264 | goat | stx2b | + | - | + | + |
| RM6933 | 84 | 96.1142 | cattle | stx1 | + | - | - | - |
| RM6934 | 84 | 96.0611 | human | stx2 | - | - | + | + |
| RM6935 | 91 | 99.0741 | food | stx1, stx2b | + | - | + | + |
| RM6936 | 91 | 96.1518 | human | stx2d | - | - | + | + |
| RM6938 | 103 | 03.2605 | horse | stx1 | + | - | - | - |
| RM6939 | 103 | 03.2606 | horse | stx1 | + | - | - | - |
| RM6940 | 103 | 03.2607 | horse | stx1 | + | - | - | - |
| RM6941 | 103 | 03.2608 | ? | stx1 | + | - | - | - |
| RM6942 | 103 | 06.1591 | ? | stx2 | - | - | + | + |
| RM6943 | 103 | 06.1623 | ? | stx2, stx2d | - | - | + | + |
| RM6944 | 111 | 00.1441 | cattle | stx1, stx2 | + | - | + | + |
| RM6945 | 111 | 00.1481 | cattle | stx1 | + | - | - | - |
| RM6947 | 111 | 00.2056 | cattle | stx1 | + | - | - | - |
| RM6949 | 113 | 95.0371 | food | stx2d | - | - | + | + |
| RM6951 | 113 | 06.1599 | ? | stx2d | - | - | + | + |
| RM6952 | 118 | 97.0167 | cattle | stx1 | + | - | - | - |
| RM6953 | 118 | 97.0192 | cattle | stx1 | + | - | - | - |
| RM6954 | 118 | 98.0556 | cattle | ~~-~~ | + | - | - | - |
| RM6955 | 121 | 00.2732 | pig | - | - | - | - | + |
| RM6956 | 121 | 07.1636 | ? | stx2 | - | - | + | + |
| RM6958 | 128 | 00.2710 | sheep | stx1a, stx2b, stx1c | + | - | + | + |
| RM6959 | 128 | 01.2446 | okapi | stx1 | + | - | - | - |
| RM6960 | 128 | 06.1595 | ? | stx2b | + | - | + | + |
| RM6964 | 145 | 95.1167 | human | stx2c | - | - | + | + |
| RM6965 | 145 | 02.3636 | rabbit | stx1 | + | - | - | - |
| RM6966 | 145 | 04.0967 | rabbit | stx2 | - | - | + | + |
| RM6967 | 145 | 04.0968 | rabbit | stx2 | - | - | + | + |
| RM6968 | 145 | 06.1598 | ? | stx2 | - | - | + | + |
| RM6969 | 153 | 02.3630 | rabbit | stx1 | + | - | - | - |
| RM6970 | 147 | 02.2555 | pig | stx1c | + | - | - | + |
| RM6973 | 147 | 02.3915 | pig | stx2e | - | - | - | + |
| RM6974 | 147 | 02.3916 | pig | stx2e | - | - | - | + |
| RM6975 | 174 | 07.1644 | ? | stx2c, stx2d | + | - | + | + |
| RM6979 | 26 | 07.3964 | ? | stx1 | + | - | - | - |
| RM6980 | 76 | 01.2265 | goat | stx1b, stx2c | + | - | + | + |
| RM6981 | 76 | 96.0893 | sheep | stx1b, stx2c | + | - | + | + |
| RM6982 | 121 | 05.0959 | ? | stx2 | - | - | + | + |
| RM6983 | 174 | 07.3964 | alpaca | stx2c | - | - | + | + |
| RM7007^e^ | 128 |  | pigeon | stx2f | - | + | - | - |
| RM6971 | 147 | 02.3788 | pig | - | - | - | - | + |
| RM6972 | 147 | 02.3789 | pig | - | - | - | - | + |

^a^ O-type was determined by ECRC.

^b^ ECRC strain number.

^c^ Genotype was determined by PCR with primers specific for stx subtypes PCR as described previously (Quinones et. al, 2012 Front. in Cell Infect. Microbiol. 2: 61).

^d^ Positive was defined as Ct < 20.

^e^ Isolated from pigeon by Schmidt et. al. 2000 Appl. Environ. Microbiol. 66:1205-1208.
